# Superelastic 3D Assembled Clay/Graphene Aerogels for Continuous Solar Desalination and Oil/Organic Solvent Absorption

**DOI:** 10.1002/advs.202205202

**Published:** 2022-11-10

**Authors:** Meichun Ding, Hao Lu, Yongbin Sun, Yujian He, Jiahui Yu, Huijun Kong, Changxiang Shao, Chen‐Yang Liu, Chenwei Li

**Affiliations:** ^1^ School of Chemistry and Pharmaceutical Engineering Shandong First Medical University & Shandong Academy of Medical Sciences Taian 271000 China; ^2^ Medical Science and Technology Innovation Center Shandong First Medical University & Shandong Academy of Medical Sciences Jinan Shandong 250117 China; ^3^ CAS Key Laboratory of Engineering Plastics CAS Research/Education Center for Excellence in Molecular Sciences Institute of Chemistry the Chinese Academy of Sciences Beijing 100190 China; ^4^ College of Materials Science and Engineering Qingdao University Qingdao 266071 China

**Keywords:** Clay/graphene aerogels, seawater desalination, solar steam generation, solvent adsorption, superelastic

## Abstract

Superelastic, arbitrary‐shaped, and 3D assembled clay/graphene aerogels (CGAs) are fabricated using commercial foam as sacrificial skeleton. The CGAs possess superelasticity under compressive strain of 95% and compressive stress of 0.09–0.23 MPa. The use of clay as skeletal support significantly reduces the use of graphene by 50%. The hydrophobic CGAs show high solvent absorption capacity of 186–519 times its own weight. Moreover, both the compression and combustion methods can be adopted for reusing the CGAs. In particular, it is demonstrated a design of 3D assembled hydrophilic CGA equipped with salt collection system for continuous solar desalination. Due to energy recovery and brine transport management promoted by this design, the 3D assembled CGA system exhibits an extremely high evaporation rate of 4.11 kg m^−2^ h^−1^ and excellent salt‐resistant property without salt precipitation even in 20 wt% brine for continuous 36 h illumination (1 kW m^−2^), which is the best reported result from the solar desalination devices. More importantly, salts can be collected conveniently by squeezing and drying the solution out of the salt collection system. The work provides new insights into the design of 3D assembled CGAs and advances their applications in continuous solar desalination and efficient oil/organic solvent adsorption.

## Graphical abstract

Superelastic, arbitrary‐shaped, and 3D assembled clay/graphene aerogels (CGAs) are fabricated using the melamine foam as a sacrificial skeleton. The resulting GCAs exhibit superelasticity, high strength, excellent flexibility, outstanding thermal stability, and tunable wettability, making them the ideal candidates for applications as promising sorbents for the removal of pollutant and high‐rate solar evaporators for continuous desalination of high‐salinity.

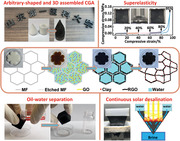

## 1 Introduction

Freshwater is the source of life and is of great significance to the survival and development of humans. Although water is the most plentiful resource on Earth, seawater accounts for ≈96.5% of the total water resources, while the proportion of freshwater resources that humans can directly consume is <0.36%.^[^ ^1^, ^2^ ^]^ With the increase in the population and improvement of living standards, freshwater water scarcity has become one of the biggest global problems.^[^ ^3^ ^]^ More than one‐third of the global population (≈2.0 billion people) lives in conditions of severe water scarcity, and this number is expected to increase to nearly 4.0 billion people by 2025.^[^ ^4^, ^5^ ^]^

Recently, many effective technologies (such as reverse osmosis, membrane distillation, and so on) have been developed to obtain freshwater from seawater.^[^ ^6^, ^7^, ^8^ ^]^ Conventional desalination technologies, which cause environmental pollution and high energy consumption, are not suitable for underdeveloped and resource‐poor areas.^[^ ^9^ ^]^ What's more, the service life of conventional equipment (such as reverse osmosis) is significantly shortened resulting from the dramatic increase in filtration pressure during continuous desalination of the high‐salinity (>7 wt.%) brine.^[^ ^10^, ^11^ ^]^ Recently, solar‐driven interfacial evaporation (SIE), which has been developed for sustainable freshwater production from seawater, has attracted extensive attention due to enhancing the evaporation rate through localizing thermal energy at the water‐air interface.^[^ ^12^, ^13^, ^14^, ^15^ ^]^ To improve solar‐driven evaporation performance, researchers have endeavored to develop highly effective SIE systems by using photothermal conversion materials such as plasmonic metallic nanoparticles,^[^ ^16^ ^]^ gel materials,^[^ ^17^, ^18^ ^]^ semiconductors,^[^ ^19^, ^20^ ^]^ polymers,^[^ ^15^, ^21^ ^]^ and carbonaceous materials.^[^ ^12^, ^22^, ^23^ ^]^ However, during the continuous desalination process, the fast steam generation at the surface of SIE device results in a significant increase in the local solute concentration, which eventually leads to the crystallization of salts on the surface. The accumulated salt in the SIE system not only seriously hinders the absorption of sunlight and reduces the light‐to‐heat conversion efficiency, but also blocks the water supply and steam release channels, resulting in a significant decrease in the evaporation performance. For SIE system, how to solve the problem of salt deposition is crucial to improve desalination performance.

Currently, there are several strategies to resolve the problem of salt accumulation. The first strategy is to construct the solar evaporators with Janus structures to generate vapor at the hydrophilic/hydrophobic interface, resulting in blocking the salt crystals.^[^ ^24^, ^25^, ^26^, ^27^ ^]^ Nevertheless, the evaporation rates of the Janus evaporators for continuous desalination are generally relatively low. Moreover, during the long‐term desalination process, due to the increase of brine salinity, it will unavoidably lead to salt deposition.^[^ ^28^, ^29^, ^30^ ^]^ The second strategy is to design solar evaporators with aligned millimeter‐scale channels that facilitate salt redissolution into the seawater through enhanced water transport.^[^ ^31^, ^32^, ^33^, ^34^ ^]^ However, the aligned channels reduce effective evaporation area and cause more energy loss to seawater, resulting in a low evaporation rate. Recently, the localized crystallization evaporator has been proposed to address salt accumulation by tuning the transport and distribution of seawater in the solar evaporator.^[^ ^35^ ^]^ The design spatially isolates the salt from the evaporation surface by facilitating radial brine transport, resulting in crystallization only at the edge of the solar evaporator.^[^ ^35^, ^36^ ^]^ However, for the localized crystallization evaporators, radial transport of seawater through 1D channels also leads to insufficient vertical absorption, reducing the evaporation performance. In addition, the bonding force between the salt crystals and the evaporation interface is very strong and only under certain conditions, such as when the slow evaporation is slow or the water content at the interface is high, the deposited salt can be partially detached by gravity.^[^ ^18^, ^35^ ^]^ If the deposited salt is not collected in time, it will inevitably lead to the reduction of the effective evaporation area and evaporation performance in the long‐term desalination process. Therefore, it remains a great challenge to construct a solar desalination system with a long‐term high and stable evaporation rate, especially when treating high‐salinity brine.

With the rapid development of industrialization, the increasing amount of industrial oily wastewater and the leakage of organic solvents have also made the problem of the freshwater shortage more serious.^[^ ^37^, ^38^, ^39^ ^]^ There is an urgent demand for materials that can effectively adsorb and separate oils and organic solvents from water, which will avoid environmental pollution and alleviate water shortages. Aerogels, as a typical 3D porous material with high porosity and low density, are considered as an ideal oil/organic solvent adsorbent.^[^ ^40^ ^]^ However, many aerogels suffer from low mechanical performance, low absorption capacity, and non‐reusability.^[^ ^41^, ^42^ ^]^ Thus, it is essential to explore an aerogel with great compressive resilience, high absorption capacity, and excellent oil‐water separation performance.

Graphene, a 2D material composed of a single layer of carbon atoms, can serve as a basic building block for the construction of 3D graphene aerogels (GAs).^[^ ^43^, ^44^, ^45^ ^]^ GAs have drawn substantial attention in the application of solar steam generation and oil/organic solvent absorption due to its low density, excellent light‐to‐heat conversion performance, hydrophobicity as well as chemical and thermal stabilities. However, the application of GAs in solar‐steam generation and oil/organic solvent absorption suffers from several limitations. Firstly, the large‐scale application of GAs is economically challenging due to the high cost of graphene. Secondly, due to the ultralow density (below 10 mg cm^−3^) of GAs, GAs are generally subject to brittle mechanical performances and the irreversible damage of network structures under working conditions. Thirdly, due to the strong hydrophobicity, GAs cannot be directly used as solar evaporators. Fourthly, GA‐based solar evaporators usually have a relatively low evaporation rate (< 2 kg m^−2^ h^−1^).

In this work, we used commercial melamine foam (MF) as sacrificial skeleton to fabricate superelastic, arbitrary‐shaped, and 3D‐assembled clay/graphene aerogels (CGAs) for solar desalination and oil/organic solvent adsorption. Clay (attapulgite (ATP), laponite (LAP), and montmorillonite (MMT)) are industrial minerals with rich reserves. Due to the advantages of low cost, good filling performance, and rich pore structure, clays can be used as adsorbents and fillers.^[^ ^46^, ^47^ ^]^ The composites made of clay and graphene through hydrogen bonds and electrostatic forces have good application prospects in the fields of composite reinforcement, pollution treatment, biocatalysis, etc.^[^ ^48^, ^49^, ^50^ ^]^ Herein, clay plays an important role in building the network of CGA and decreases the use of graphene by 50%, resulting in reducing the cost. The resulting CGAs show superelasticity with a compressive stress of 0.09–0.23 MPa and a compressive strain of 95%. The hydrophilicity and hydrophobicity of CGAs (the contact angle to water was 56–141°) can be conveniently tuned by introducing different kinds of clay. The hydrophobic CGAs show a high oil/organic solvent absorption capacity of 186–519 times its own weight. The oil–water separation performance can be effectively improved by constructing 3D‐assembled CGAs. Moreover, CGAs exhibit high and stable adsorption capacity in cyclic use due to the robust network structures. In addition, it is demonstrated that 3D assembled hydrophilic CGA equipped with a salt collection system (pre‐pressed MF, denoted as p‐MF) can serve as a high‐rate and continuous solar evaporator for high‐salinity brine desalination. Due to energy recovery and brine transport management promoted by this design, the 3D assembled CGA system exhibits an extremely high steam generation rate of 4.11 kg m^−2^ h^−1^ and excellent salt‐resistant properties without salt precipitation in 20 wt.% brine for continuous 36 h illumination (1 kW m^−2^). Salts can be collected conveniently by periodically squeezing and drying the solution out of the p‐MF. Remarkably, even after over one week floating in the 20 wt.% brine, the high evaporation rate is successfully maintained without salt deposition. The 3D‐assembled CGAs hold great potential for applications in continuous solar desalination of high‐salinity and highly efficient oil/organic solvent adsorption.

## 2 Results and Discussion

### 2.1 Fabrication and Characterization of CGAs

**Figure 1:**
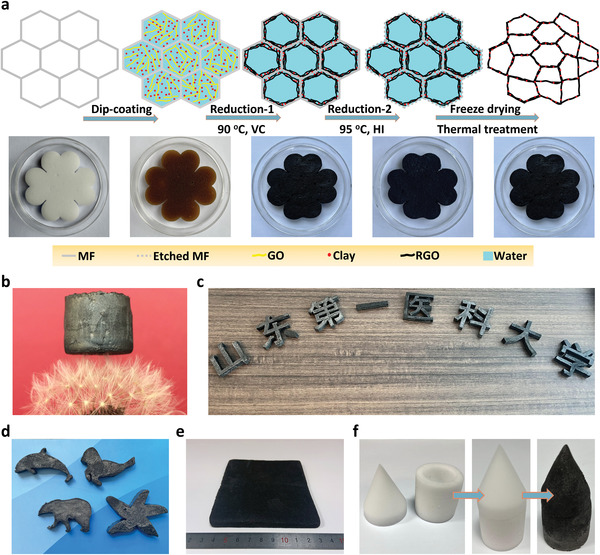
a) Schematic showing the fabrication process of CGA. b) Photograph of a CGA (≈ 4 mg cm^−3^) placed on a dandelion. Photographs of CGAs of Chinese character of c) “
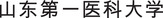
” for Shandong First Medical University, d) “dolphin”, “sea lion”, “polar bear”, and “starfish”. e) The photograph of large‐scale CGA. f) Photographs of 3D large CGA assembled using the cup‐shaped MF and conical MF as sacrificial templates.

The schematic of the fabrication process of CGA is shown in **Figure** **1**a and the detailed preparation process is described in the experimental section. The as‐prepared ATP, LAP, and MMT can be dispersed in water to form a stable aqueous suspension. The clay (ATP, LAP, and MMT) suspensions and graphene oxide (GO) suspension were mixed to obtain uniform mixed suspensions, respectively (Figures S1 and S2, Supporting Information). According to the weight ration of clay (ATP, LAP, and MMT) and GO as 1:1, the ATP/graphene aerogels, LAP/graphene aerogels, and MMT/graphene aerogels obtained from the mixed solutions are referred to as AGAs, LGAs, and MGAs, respectively. The commercial melamine foam (MF) with excellent processability, and a low‐cost 3D interconnected polymer network (Figure S3, Supporting Information), was chosen as the sacrificial template to fabricate CGAs. Herein, a piece of MF was cut into a cloud shape as an example to demonstrate the synthesis process of a cloud‐shaped CGA (Figure 1a). Ascorbic acid (VC) was employed as a reducing agent and mixed with clay/GO suspension under vigorous stirring. The cloud‐shaped MF was immersed in the above‐mixed suspension and then squeezed several times. The clay/GO/MF composite was placed in an oil bath (90 °C) for 4 h to obtain clay/reduced GO (RGO)/MF hydrogel (reduction‐1 process). RGO sheets self‐assembled into network structures in the skeleton of MF (Figure S4, Supporting Information). The porous skeleton of MF with the pore size of ≈50–150 µm could effectively prevent the RGO sheets from restacking via the enhanced *π*–*π* interactions during the reduction‐1 process. In contrast, without MF as a sacrificial skeleton, RGO sheets self‐assembled into denser network structures (Figure S5, Supporting Information), resulting in severe volume shrinkage of clay/RGO hydrogel (Figure S6, Supporting Information). The resultant clay/RGO/MF hydrogel was placed in hydroiodic acid solution (HI, 37%) at 95 °C for 4 h to further eliminate the oxygen‐containing functional groups on RGO surface (reduction‐2 process) and remove the MF skeleton.^[^ ^51^ ^]^ The obtained clay/RGO hydrogel was washed with water to remove the residual impurities, followed by freeze drying and thermal treatment to obtain a CGA.

The densities of CGAs can be tuned from 2 to 6 mg cm^−3^ by manipulating the concentration of GO solutions from 3 to 5 mg mL^−1^ (Figure S7, Supporting Information). A CGA with the density of ≈4 mg cm^−3^ and a volume of ≈3 cm^3^ could stand on a dandelion without any deformation of the soft fluff (Figure 1b). As shown in Figure 1a, the obtained CGA almost retained the cloud shape of MF. Therefore, CGAs could be fabricated into arbitrary‐shapes using the MF with pre‐designed shapes, such as the Chinese character of “
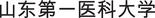
” for Shandong First Medical University, “dolphin”, “sea lion”, “polar bear”, and “starfish” (Figure 1c,d). The convenient method could easily use large‐sized MF as a sacrificial template to fabricate large‐scale CGA (Figure 1e). More importantly, a large 3D CGA could be prepared by using several small MFs, such as assembling cup‐shaped MF and conical MF into a 3D assembled CGA (Figure 1f). As shown in Figure S8 (Supporting Information), there must be large interface gaps when the three short MFs were placed together. The gaps between adjacent MFs were completely repaired after being assembled into a 3D CGA (Figure S9, Supporting Information). Therefore, 3D‐assembled CGAs with different functions and shapes can be flexibly integrated according to the different applications.

**Figure 2:**
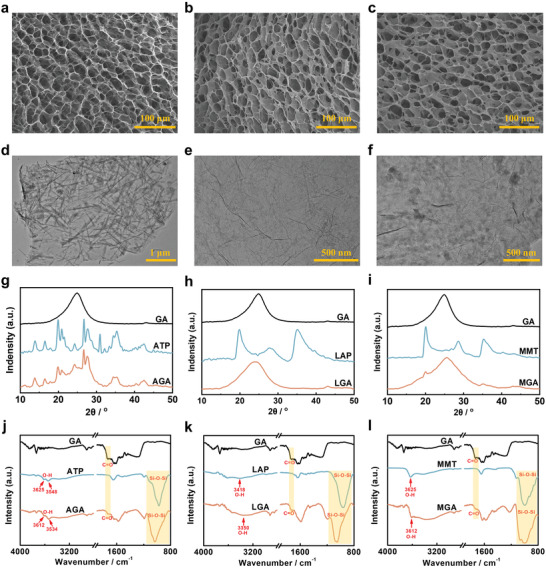
SEM images of a) AGA, b) LGA, and c) MGA. TEM images of d) AGA, e) LGA, and f) MGA. XRD patterns of g) AGA, h) LGA, and i) MGA. FTIR spectrums of j) AGA, k) LGA, and l) MGA.

During the freezing process, the clay/RGO sheets were forced to align along the growth direction of ice crystals, resulting in forming a 3D interconnected clay/RGO network structure. As shown in **Figure** **2**a–c, Figure S10, and Table S1 (Supporting Information), CGAs and GA showed similar honeycomb‐like porous structures with a pore size of several tens of micrometers and surface areas of 15–69 m2 g^−1^. Moreover, the scanning electron microscopic (SEM) image showed that CGA (taking AGA as an example, shown in Figure S11, Supporting Information) exhibited a large‐area (>1 mm^2^) uniform porous structure, which is beneficial for the elasticity of aerogels.^[^ ^52^, ^53^ ^]^ The transmission electron microscopy (TEM) images of AGA, LGA, MGA, and GA are shown in Figure 2d–f and Figure S12 (Supporting Information), respectively. The TEM images of AGA and MGA revealed that the ATP and MMT were well dispersed on the graphene sheets. For LGA, it is difficult to observe LAP on RGO sheets due to the small size and thickness of LAP (Figure S1, Supporting Information). According to the SEM elemental mapping images (Figure S13, Supporting Information), it can be seen the Mg and Si elements were well distributed on the graphene sheets, confirming that LAP was homogeneously dispersed into the network structure of LGA. In addition, wrinkle regions can be observed on the surfaces of RGO sheets of AGA, LGA, and MGA, which are beneficial to improve the resilience performance of aerogels (Figure 2d–f).^[^ ^54^ ^]^ Furthermore, the X‐ray diffraction (XRD) patterns of CGA, GO, and GA are shown in Figure 2g–i and Figure S14 (Supporting Information), respectively. The GO and GA show the XRD diffraction peaks at 11.5^o^ and 24.6^o^, indicating that the enhanced *π*–*π* interactions between RGO sheets due to the removal of oxygen‐containing functional groups. AGA and MGA showed the characteristic diffraction peaks for ATP and MMT, indicating that ATP and MMT were loaded into the 3D network structures of AGA and MGA, respectively. LGA showed one wide peak at ≈25^o^, and the reason should be that LAP was peeled off and distributed into the network structure of LGA in a disordered state.

The chemical compositions of the AGA, LGA, MGA, and GA were analyzed by Fourier transform infrared (FTIR) spectroscopy (Figure 2j–l), respectively. The spectrum of GA shows absorption signals at ≈1738 cm^−1^, which can be attributed to the carbonyl C=O.^[^ ^55^ ^]^ The spectrums of ATP, LAP, and MMT show the absorption signal at 962–993 cm^−1^, corresponding to the Si—O—Si stretching vibration.^[^ ^56^, ^57^, ^58^, ^59^ ^]^ All the characteristic peaks of CGAs and GA appear in the FTIR spectra of an AGA, LGA, and MGA (Figure 2j–l), confirming the presence of ATP, LAP, and MMT in the network structure, respectively. Compared with the signal from ATP, LAP, and MMT, the absorption band of the O‐H stretching vibration in an AGA, LGA, and MGA shifted from 3418–3625 to 3350–3612 cm^−1^, indicating that H‐bonding interactions formed between ATP, LAP, MMT, and RGO sheets, respectively. Thermal gravimetric analysis (TGA) curves are shown in Figure S15 (Supporting Information), and CGAs have better thermal stability compared to GA.

### 2.2 Mechanical Properties of CGAs

**Figure 3:**
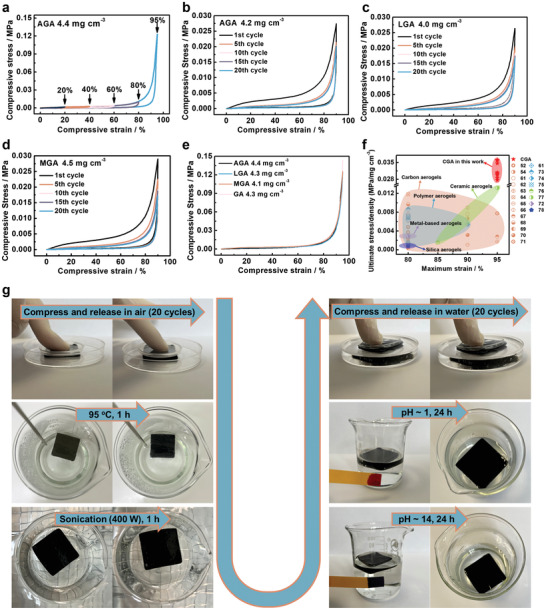
a) The stress‐strain curves of AGA at different compressive strains. The compressive stress‐strain curves of b) AGA, c) LGA, and d) MGA for 20 cycles of loading and unloading. e) Stress–strain curves of CGAs and GA at strain of 95%. f) Comparison of the normalized ultimate stress (compressive stress divided by density) of CGAs with other elastic aerogels as a function of the maximum strain. The detailed data for all the referred samples are listed in Table S3 (Supporting Information). g) The durability and stability of an AGA under a series of continuous harsh tests: 20 cycles of compression test in air, high temperature (95 °C, 1 h), ultrasonic agitation (400 W, 1 h), alkaline (pH ≈ 14, 24 h) environment, acidic (pH ≈ 1, 24 h) environment, and 20 cycles of compression test in water.

Excellent mechanical strength is essential for solar steam generation devices and oil/organic solvent absorbents to maintain structural and performance stability under long‐term use processes. Especially for low‐density GA, the graphene network structure is prone to irreversible damage due to dynamic seawater/solvent flow and extrusion under practical working conditions.^[^ ^60^ ^]^ We investigated the compression behaviors of AGA, LGA, and MGA, respectively. The cyclic compression tests of AGA with the increasing strains (20%, 40%, 60%, 80%, and 95%) are shown in **Figure** **3**a and Movie S1 (Supporting Information). The AGA with a density of 4.4 mg cm^−3^ exhibited compressive stress of 0.122 mPa at 95% strain and fully recovered to its original height after the force was released. As shown in Figure 3b–d, the AGA (4.2 mg cm^−3^), LGA (4.0 mg cm^−3^), and MGA (4.5 mg cm^−3^) showed excellent fatigue‐resistant performance and could fully recover to their original heights after they were compressed to 90% strain for 20 cycles. The compressive stress‐strain curves of CGAs showed three regimes during the compression process: linear‐elasticity (*ε* < ≈15%), plateau (≈15% < *ε* < ≈70%), and densification (≈70% < *ε*).^[^ ^61^ ^]^ Compared to the GA (4.2 mg cm^−3^), the AGA, LGA, and MGA showed slightly smaller compressive stress of 0.122, 0.123, and 0.125 mPa, respectively (Figure 3e; Table S2, Supporting Information). Compared with other elastic aerogels (density ranges from 0.56 to 31.20 mg cm^−3^), such as carbon‐based aerogels,^[^ ^52^, ^54^, ^61^, ^62^, ^63^, ^64^, ^65^, ^66^, ^67^, ^68^, ^69^, ^70^, ^71^ ^]^ metal‐based aerogels,^[^ ^72^ ^]^ polymer‐based aerogels,^[^ ^61^, ^73^, ^74^, ^75^ ^]^ ceramic aerogels,^[^ ^76^, ^77^ ^]^ and silica aerogels,^[^ ^78^ ^]^ CGAs can endure the largest compressive strain and possess the highest normalized compressive stress density (Figure 3f; Figures S16 and S17, and Table S3, Supporting Information). Therefore, the excellent elasticity and fatigue resistance of CGAs can keep the network structure intact under dynamic seawater/solvent flow and extrusion. Moreover, to evaluate the stability of CGA in harsh conditions, an AGA was continuously undergone a series of harsh conditions, including 20 cycles ofcompression and releasing in air, high temperature (≈95 °C, 1 h), ultrasonic agitation (400 W, 1 h), alkaline environment (pH ≈14, 24 h), acidic environment (pH ≈1, 24 h), and cyclic compression and releasing in water (20 cycles). After these tests, the appearance and porous network structures of AGA remained almost unchanged, indicating its durability and excellent structural stability (Figure S18, Supporting Information). Therefore, CGA can maintain structural stability under long‐term harsh environments, which is essential for achieving long‐term durability and reusability.

### 2.3 The Tunable Wettability and Adsorption Performance of CGAs

**Figure 4:**
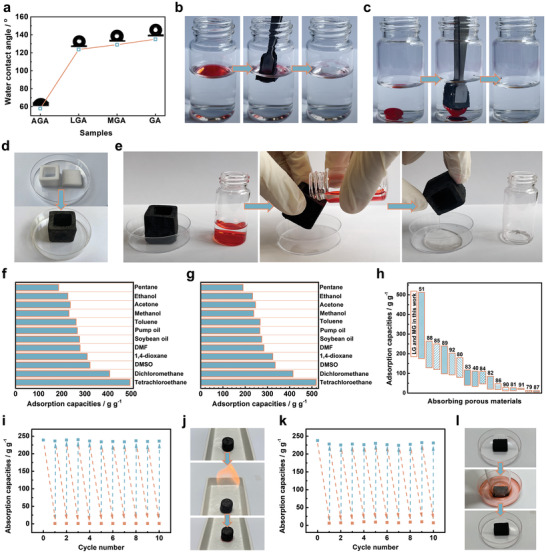
a) The contact angles of CGAs and GA. Snapshots of fast absorption of b) toluene (dyed with Sudan III) floating on water and c) dichloromethane (dyed with Sudan III) on the bottom of water using LGA and MGA, respectively. d) Photographs of the assembly process of 3D cubic cup‐shaped CGA. e) Fast separation of dichloromethane (dyed with Sudan III) and water with the 3D cubic cup‐shaped MGA with within seconds. Absorption capacities of g) LGA and (h) MGA for various organic solvents and oils. i) Absorption performance stability of MGA for ethanol during cyclic absorption/combustion process. j) Snapshots of the process for recycling MGA via combustion. k) Absorption performance stability of MGA for ethanol during cyclic absorption/compression process. l) Snapshots of the process for recycling MGA via compression.

Hydrophilic and hydrophobic properties are important factors affecting the adsorption performance, the wettability of CGA was evaluated by water contact angle measurement. The LGA and MGA showed the water contact angle of 123.7^o^ and 129.0^o^, which were slightly lower than the 135.1^o^ of GA, showing remarkable hydrophobicity properties (**Figure** **4**a). Due to their low density, highly porous structure (porosity: ≈99.6%), and hydrophobicity property, LGA and MGA exhibited excellent adsorption capability. As shown in Figure 4b,c and Movies S2 and S3 (Supporting Information), LGA and MGA could completely absorb the toluene (top; dyed with Sudan III) and chloroform (bottom; dyed with Sudan III) from water within ≈4 s, respectively. Moreover, thanks to the excellent processability of CGA (Figure 1f), the construction of a 3D‐assembled CGA can be easily achieved. Herein, the cubic cup‐shaped MGA was chosen as an example to illustrate that 3D assembled CGA could effectively improve oil‐water separation performance (Figure 4d). As shown in Figure 4e and Movie S4 (Supporting Information), when the dichloromethane (dyed with Sudan III)/water mixture was poured into the cubic cup‐shaped MGA, the dichloromethane was rapidly absorbed, resulting in complete separation of water and dichloromethane within several seconds. Similarly, the toluene (dyed with Sudan III)/water mixed solution could be effectively separated by the cubic cup‐shaped MGA (Figure S19 and Movie S5, Supporting Information).

To study the absorption capacity of LGA and MGA, multiple kinds of organic solvents were chosen, such as common solvents (acetone, 1,4‐dioxane, dimethylformamide, and Dimethyl sulfoxide), alcohols (methanol and ethanol), aromatics (dichloromethane and tetrachloroethane), alkanes (pentane and toluene), and commercial oil products (pump oil and soybean oil). As shown in Figure 4f,g, Figure S20, and Table S4 (Supporting Information), LGA and MGA showed high absorption capacity similar to GA, 186–495 and 190–519 times its own weight depending on the solvent type and density, respectively. Moreover, the absorption capacities of LGA and MGA are higher than that of many reported porous materials, such as inorganic‐based aerogels (silicone sponge),^[^ ^79^ ^]^ carbon‐based aerogels (CNT aerogels, graphene aerogels, carbon aerogels, and so on),^[^ ^40^, ^51^, ^80^, ^81^, ^82^, ^83^, ^84^, ^85^, ^86^, ^87^, ^88^, ^89^ ^]^ and polymer‐based aerogels (cellulose aerogels, PU foams, and so on)^[^ ^90^, ^91^, ^92^ ^]^ (Table S5, Supporting Information). Therefore, the LGA and MGA show great potential in practical applications for the removal of pollutants.

In practical applications, most organic pollutants from industry are valuable or noxious, the collection of pollutants and recyclability of adsorbents are very important. There are two methods of recycling CGAs depending on the type of solvent. Combustion is a simple method to remove adsorbed toxic or low‐value solvents. The recycling test of MGA was performed using the cyclic absorption‐combustion method (Figure 4i). As shown in Figure 4j and Movie S6 (Supporting Information), MGA could keep its initial shape after cyclic absorption/combustion tests. The robust network structure and excellent thermal stability endow MGA with high and stable adsorption capacity. Thanks to the excellent elasticity of CGA, compression is an ideal choice to recover valuable solvents. As shown in Figure 4k,l and Movie S7 (Supporting Information), MGA maintained the high adsorption capacity and its original shape during the cyclic adsorption‐compression tests. Therefore, the CGA exhibits remarkable potential as an ideal material for recovering solvents and collecting organic pollutants.

### 2.4 3D Assembled CGA for High‐Rate and Continuous Solar Desalination

**Figure 5:**
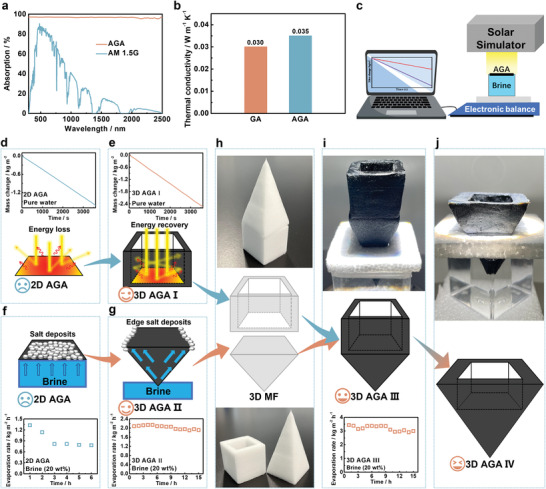
a) Absorption spectrum of AGA and air mass 1.5 global (AM 1.5 G) tilt solar spectrum. b) Thermal conductivities of GA and AGA. c) Schematic illustration for the experimental setup of the solar steam generation test. Schematic showing of d) energy loss of 2D AGA and e) energy recovery of 3D AGA I. Insert: The mass change of water versus time with 2D AGA and 3D AGA I under one sun illumination, respectively. Schematic illustration of brine transport and salt crystallization performance of f) 2D AGA and g) of 3D AGA II. Insert: The evaporation rates of 2D AGA and 3D AGA II in 20 wt.% brine under one sun illumination, respectively. h,i) Schematic illustration of the assembly process of 3D AGA III by using a pyramidal MF and a cubic‐cup‐shaped MF. Insert: The evaporation rates of 3D AGA III in 20 wt.% brine under one sun illumination. j) Schematic illustration of the design of 3D AGA IV with the expanding cross‐section perpendicular to brine flow (Figure S28, Supporting Information).

Humanity is facing an increasingly serious problem of freshwater scarcity. Abundant and clean solar energy makes solar desalination one of the most promising techniques to obtain freshwater from seawater. AGA showed excellent processability (Figure 1), excellent mechanical properties (Figure 3), high porosity (porosity: ≈99.6%. surface area: 69 m2 g^−1^, shown in Table S2, Supporting Information), excellent hydrophilic properties (Figure 4a), excellent solar absorption (**Figure** **5**a), and low thermal conductivity (Figure 5b), thus AGA has great potential for high‐rate solar evaporation. As shown in Figure 5c, the solar evaporation performance of AGA was evaluated by using electrical balance under the simulated sunlight illumination. Brine was efficiently transported to the top of AGA by capillary action due to the abundant porous structure (Figure S21, Supporting Information). The schematic of the design process of the AGA‐based solar evaporator for continuous desalination is shown in Figure 5d–j.

As shown in Figure 5d, 2D AGA showed an evaporation rate of 1.62 kg m^−2^ h^−1^ under one sun illumination. The low evaporation rate should be attributed to the energy loss caused by the diffuse reflection and thermal radiation of 2D AGA under illumination.^[^ ^93^, ^94^ ^]^ Compared to the 2D solar evaporator, the 3D cup‐shaped one showed a higher evaporation rate because its walls could reabsorb the diffuse reflection and thermal radiation from the bottom (Figure 5e).^[^ ^94^ ^]^ Moreover, 3D cup‐shaped solar evaporators could also gain additional energy from the ambient environment. Herein, a 3D cubic cup‐shaped AGA (denoted as 3D AGA I) could be easily synthesized due to the excellent processability of MF (Figure S22, Supporting Information). In contrast, the evaporation rate of 3D AGA I was 2.52 kg m^−2^ h^−1^ under one sun illumination (Figure 5e).

The highly concentrated artificial brine (20 wt.% NaCl) was used for solar desalination tests. Due to the lower water vapor pressure of 20 wt.% brine, 2D AGA showed an evaporation rate of 1.34 kg m^−2^ h^−1^ under one sun illumination, which was slightly smaller than that of pure water (1.62 kg m^−2^ h^−1^) (Figure 5f). After ≈1.5 h of continuous desalination, the evaporation rate gradually decreased due to the obvious salt accumulation on the surface of 2D AGA (Figure S23, Supporting Information). To solve the salt deposition problem, we prepared a pyramidal AGA (denoted as 3D AGA II) with an expanding cross‐section perpendicular to the brine flow direction, which facilitated radial brine transport, resulting in edge‐preferential salt crystallization (Figure 5g; Figure S24, Supporting Information).^[^ ^18^ ^]^ Due to edge‐preferential salt crystallization, the average evaporation rates of 3D AGA II could be maintained at ≈2.00 kg m^−2^ h^−1^ during continuous 15 h desalination process (Figure 5g; Figure S24, Supporting Information).

Compared with 2D AGA, the 3D AGA I and 3D AGA II achieved higher and more stable evaporation rates during continuous desalination through energy recovery and edge‐preferential salt crystallization, respectively. Therefore, high‐rate evaporation and edge‐preferred salt crystallization could be achieved simultaneously by combining pyramidal AGA and cubic cup‐shaped AGA into one large 3D AGA (denoted as 3D AGA III) (Figure 5h,i). Under one sun illumination for 15 h continuous desalination, the average evaporation rate of the 3D AGA III is ≈3.20 kg m^−2^ h^−1^ (Figure 5i). After the desalination test, salt accumulation occurred on the upper surface of the 3D AGA III, while no salt deposition occurred on the bottom and walls of the cubic‐cup part (Figure S25, Supporting Information). However, due to the cubic‐cup part of the 3D AGA III is the same in a cross‐section perpendicular to brine flow, the salt gradually accumulated on the cup walls under three sun illumination, resulting in a decrease in the effective evaporation area (Figure S26, Supporting Information). Therefore, we further improved the design of 3D AGA III to propose the 3D AGA IV, whose cup part had the expanding cross‐section perpendicular to brine flow, resulting in edge‐preferential salt crystallization (Figure 5j; Figures S27 and S28, Supporting Information). As shown in Figure S29 (Supporting Information), the average evaporation rates of 3D AGA IV are 2.72, 3.59, 4.37, 5.27, and 6.85 kg m^−2^ h^−1^ in 20 wt.% brine under 0.5, 1, 1.5, 2, and 3 sun illuminations, respectively. The 3D AGA IV could simultaneously achieve solar steam generation and salt collection due to energy recovery, ambient energy harvesting, and edge‐preferential crystallization. However, due to the strong binding force between salt crystals and the evaporation interface, only when the evaporation rate was slow or the water content of the evaporation interface increased, the deposited salt could be partially detached under the effect of gravity.^[^ ^18^, ^35^ ^]^ How to collect salt conveniently and timely is a difficult problem, especially for large desalination devices composed of several solar evaporators. More importantly, if the deposited salt is not removed in time, it will inevitably lead to the reduction of the effective evaporation area and evaporation rate. It is still a great challenge to construct an ideal solar desalination system with high‐rate and continuous evaporation, no salt deposition, and convenient salt collection.

**Figure 6:**
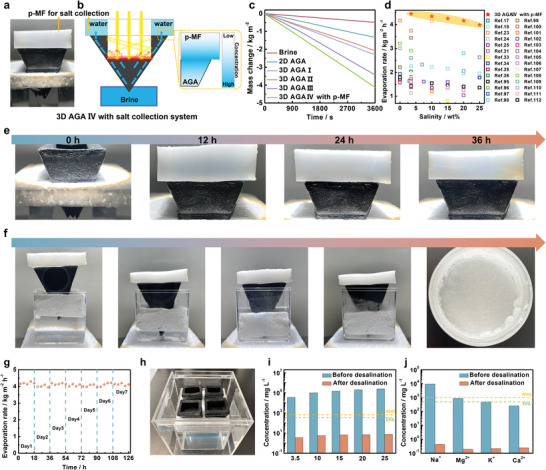
a) Photograph of and b) schematic illustration of 3D AGA IV equipped with p‐MF as salt collection system: a piece of p‐MF full of water was located on the upper surface of the 3D AGA IV as salt collection system. The p‐MF had a hole with the same size as the inner diameter of the 3D AGA IV to allow sunlight to pass through. Due to the increasing cross‐section area of 3D AGA IV, the brine transport was along the radial, resulting in the increase of brine concentration at the edge of the upper surface of 3D AGA IV. In this way, the p‐MF acted as salt collection system to absorb the salt from upper surface of the 3D AGA IV driven by the salt concentration gradient. c) The mass change of 20 wt.% brine versus time with 2D AGA and 3D AGA I – IV under one sun. d) Solar evaporation performance of the p‐MF‐equipped 3D AGA IV in brine with different salinity (3.5–25 wt.%) compared with recent literature reports. e) Photographs of the p‐MF‐equipped 3D AGA IV at 0, 12, 24, and 36 h during the continuous desalination test under 1‐sun illumination, respectively. No salt accumulation was observed on the inner surface and outer surface of the 3D AGA IV during the continuous desalination process (Figure S37, Supporting Information). f) Time‐dependent photographs of the p‐MF‐equipped 3D AGA IV for continuous solar desalination. Photographs of collected salt are presented on the right. Complete separation of water and salt after solar desalination. g) Long‐term evaporation rate of the p‐MF‐equipped 3D AGA IV in 20 wt.% brine under one sun illumination for seven days with 18 h every day. h) Photograph of the 2 × 2 arrays of the 3D AGA IV (equipped with p‐MF) floating on the brine in a freshwater collection device (Figure S42, Supporting Information). i) Measured concentration of Na^+^ in brine with different salinity (3.5–25 wt.%) before and after solar desalination. j) The measured concentrations of four primary ions in a real seawater sample (from Hongdao, Qingdao, China) before and after solar desalination.

To solve the salt deposition and salt collection issues, a piece of pre‐pressed MF (referred as p‐MF) full of water was placed on upper surface of the 3D AGA IV as salt collection system (**Figure** **6**a). The p‐MF had a hole with the same size as the inner diameter of the 3D AGA IV to allow sunlight to pass through (Figure S30, Supporting Information). The p‐MF was fabricated by hot‐pressing treatment of the pristine MF (Figure S31, Supporting Information). Compared to the pristine MF, p‐MF had a denser flexuous network with a smaller pore size (Figure S32, Supporting Information), resulting in better fluidic transport properties (Figure S33, Supporting Information). Due to the increased cross‐section area of 3D AGA IV, the brine transport was along the radial, resulting in the increase of brine concentration on the edge (Figure S28, Supporting Information). The dense and flexuous network constructed via the p‐MF could absorb the salt from upper surface of the 3D AGA IV driven by the salt concentration gradient (Figure 6b; Figure S34, Supporting Information). Thanks to the salt collection of the p‐MF, the 3D AGA IV showed an extremely high steam generation rate of 4.13 kg m^−2^ h^−1^ (Figure 6c). Note that the evaporation rates of all 3D AGA IV equipped with p‐MF in this work had been calculated by subtracting the evaporation rate of p‐MF under one sun illumination. Moreover, the evaporation enhancement is also attributed to the harvesting of ambient energy (Figure S35, Supporting Information). As shown in Figure 6d and Table S6 (Supporting information), the evaporation performances of the 3D AGA IV are superior to those of recently reported solar generation systems^[^ ^17^, ^18^, ^23^, ^24^, ^25^, ^31^, ^33^, ^34^, ^35^, ^36^, ^95^, ^96^, ^97^, ^98^, ^99^, ^100^, ^101^, ^102^, ^103^, ^104^, ^105^, ^106^, ^107^, ^108^, ^109^, ^110^, ^111^, ^112^ ^]^ under a wide salinity brine (3.5–25 wt%). To investigate the no salt deposition and salt collection behavior of the p‐MF‐equipped 3D AGA IV when treating the high‐salinity brine, a continuous 36 h desalination test was performed in 20 wt.% brine under one sun illumination. As shown in Figure 6e and Figures S36 and S37 (Supporting Information), 3D AGA IV equipped with p‐MF showed a high average evaporation rate of ≈4.11 kg m^−2^ h^−1^ and no salt accumulation was observed on the inner and outer surface of the 3D AGA IV for a continuous 36 h illumination (one sun). To evaluate the salt collection of p‐MF, the solution was squeezed from p‐MF every 9 h and dried to evaluate the weight of the collected salt (Figures S38 and S39, Supporting Information). More importantly, with the evaporation of water, the p‐MF‐equipped 3D AGA IV floating on the brine surface gradually moved down during the continuous solar desalination process, finally achieving the complete separation of salt and water (Figure 6f).

For practical applications, the durability of a solar desalination system is a critical factor to consider. The p‐MF‐equipped 3D AGA IV floated on the surface of 20 wt.% brine for 7 days and was then irradiated under one sun irradiation for a continuous 18 h per day. As shown in Figure 6g, the p‐MF‐equipped 3D AGA IV was found to maintain a stable and high evaporation rate (≈4.10 kg m^−2^ h^−1^) over one week, showing no precipitated salt (Figure S40, Supporting Information). After the durability tests, the 3D AGA IV was washed in the water for several times and then dried and characterized. According to the SEM image (Figure S41, Supporting Information), it was found that the porous structure of the 3D AG IV remained unchanged, indicating excellent structural stability and durability.

For a solar desalination system, the salinity level of the evaporated water is another important factor to evaluate the effect of desalination.^[^ ^113^ ^]^ As shown in Figure 6h, we set up a 2 × 2 array of the 3D AGA IV floating on the brine in a chamber and outer container is the freshwater collection chamber. The whole device was then sealed in a transparent house‐like acrylic board (Figure S42, Supporting Information). The steam condensed on the transparent acrylic board to form fresh water which can flow into the container for collection. The concentration of Na^+^ was significantly reduced after desalination of a wide salinity brine with the 3D AGA IV and were much below the drinking water standard defined by World Health Organization (WHO) and the US Environmental Protection Agency (EPA) (Figure 6i and Table S7, Supporting information). Furthermore, real seawater from Hongdao, Qingdao, China was used for solar desalination via the 3D AGA IV. The concentrations of Ca^2+^, K^+^, Na^+^, and Mg^2+^ significantly decreased with the ion rejection ratio of ≈99% after desalination (Figure 6j; Table S8, Supporting Information). Therefore, above results demonstrate that the solar evaporation device based on the 3D AGA IV can effectively purify a wide salinity brine (3.5–25 wt.%), indicating great potential in practical seawater desalination applications.

## 3 Conclusion

Herein, superelastic, arbitrary‐shaped, and 3D assembled clay/graphene aerogels (CGAs) are fabricated using commercial foam as a sacrificial skeleton. The CGAs possess superelasticity under the compressive strain of 95% and compressive stress of 0.09–0.23 MPa. The use of clay as skeletal support significantly reduces the use of expensive graphene by 50%. The hydrophobic CGAs show a high organic solvent absorption capacity of 186–519 times its own weight. Moreover, both compression and combustion methods can be adopted for reusing the CGAs. In particular, it demonstrated a design of a 3D assembled hydrophilic CGA equipped with a salt collection system for high‐rate and continuous solar desalination of high‐salinity. Due to energy recovery and brine transport management promoted by this design, the 3D assembled CGA system exhibits an extremely high evaporation rate of 4.11 kg m^−2^ h^−1^ and excellent salt‐resistant property without salt precipitation even in 20 wt.% brine for continuous 36 h illumination (1 kW m^−2^), which is the best‐reported result from the solar desalination devices. More importantly, salts can be collected conveniently by periodically squeezing and drying the solution out of the salt collection system, finally achieving the complete separation of salt and water. The work provides new insights into the design of 3D assembled CGAs and advances their applications in continuous solar desalination and highly efficient oil/organic solvent adsorption.

## 4 Experimental Section

Experimental details are given in the Supporting Information.

## Conflict of Interest

The authors declare no conflict of interest.

## Supporting information

Supporting Information

Supplemental Movie 1

Supplemental Movie 2

Supplemental Movie 3

Supplemental Movie 4

Supplemental Movie 5

Supplemental Movie 6

Supplemental Movie 7

## Data Availability

The data that support the findings of this study are available from the corresponding author upon reasonable request.
