# Supplementary material for: Superelastic 3D Assembled Clay/Graphene Aerogels for Continuous Solar Desalination and Oil/Organic Solvent Absorption
Source: Adv Sci (Weinh). 2022 Nov 10;9(36):2205202. doi: 10.1002/advs.202205202 (PMC9798983; doi:10.1002/advs.202205202)
Supplement: Supplementary file 1 — Supporting Information [file ADVS-9-2205202-s001.pdf]

ADVANCED SCIENCE
Open Access

Supporting Information

for *Adv. Sci.*, DOI 10.1002/advs.202205202

Superelastic 3D Assembled Clay/Graphene Aerogels for Continuous Solar Desalination and Oil/Organic Solvent Absorption

*Meichun Ding, Hao Lu, Yongbin Sun, Yujian He, Jiahui Yu, Huijun Kong, Changxiang Shao, Chen-Yang Liu and Chenwei Li**

Supporting Information

Superelastic 3D assembled clay/graphene aerogels for continuous solar desalination and oil/organic solvent absorption

Meichun Ding,[ab] *Hao Lu,*[c] *Yongbin Sun,*[a] *Yujian He,*[d] *Jiahui Yu,*[b] *Huijun Kong,*[ab] *Changxiang Shao,*[ab] *Chen-Yang Liu,*[c] *Chenwei Li*[*ab]

[a] School of Chemistry and Pharmaceutical Engineering, Shandong First Medical University & Shandong Academy of Medical Sciences, Taian 271000, China.

[b] Medical Science and Technology Innovation Center, Shandong First Medical University & Shandong Academy of Medical Sciences, Jinan 250117, China

[c] CAS Key Laboratory of Engineering Plastics, CAS Research/Education Center for Excellence in Molecular Sciences, Institute of Chemistry, the Chinese Academy of Sciences, Beijing 100190, China.

[d] College of Materials Science and Engineering, Qingdao University, Qingdao 266071, China.

[*] Corresponding Author: lichenwei@iccas.ac.cn (C. W. Li).

Contents

1. Supplementary Figures and Tables...5

1.1 TEM Images of ATP, MMT, and LAP 5

1.2 Atomic Force Microscopy (AFM) Image of GO 6

1.3 SEM Images of MF 7

1.4 SEM Image of MF/RGO/Clay Composite 8

1.5 SEM Images of GA Prepared Without Using MF as Skeleton 9

1.6 Photos of Clay/GO Suspension and Clay/RGO Hydrogel. 10

1.7 The Densities of CGAs 11

1.8 Schematic Diagram of Gap Repair During 3D CGA Assembly Process......... 12

1.9 SEM Image of the Gap Junction Cross-section of a 3D Assembled CGA 13

1.10 SEM Image of GA... 14

1.11 Specific Surface Area of GA and CGAs 15

1.12 SEM Image of AGA with Low Magnification.. 16

1.13 TEM Images of GO and GA 17

1.14 SEM Elemental Mapping Images of LGA 18

1.15 XRD Patterns of GO and GA.. 19

1.16 TGA curves of GA and CGAs 20

1.17 The Compression Performances of GA and CGAs....................................... 21

1.18 The Compression Performances of CGAs with Different Densities............. 22

1.19 Comparison of the Compression Performances of CGAs with Other Elastic Aerogels 23

1.20 Comparison of the Compression Performances of CGAs with Other Elastic Porous Materials.. 24

1.21 SEM Image of AGA After a Series of Harsh Tests 26

1.22 Fast Separation of Organic Solvent/Water with 3D Assembled MGA.......... 27

1.23 Adsorption Capacities of the GA 28

1.24 Adsorption Capacities of the LGA, MGA, and GA...................................... 29

1.25 Comparison of Adsorption Capacities of CGAs and other Porous Materials Reported in the Literatures.. 30

1.26 Schematic Illustration for The Experimental Setup of The Solar Steam Generation Test 31
1.27 The Consturction of 3D AGA I 32
1.28 Continuous Desalination Test for 2D AGA 33
1.29 Continuous Desalination Test for 3D AGA II 34
1.30 Continuous Desalination Test for the 3D AGA III 35
1.31 Schematic Illustration of the Continuous Desalination Test for the 3D AGA III Under 1 and 3 Sun Illumination 36
1.32 The Consturction of the 3D AGA IV 37
1.33 Schematic Illustration of the Continuous Desalination Test for the 3D AGA IV Under 3 Sun Illumination 38
1.34 The Evaporation Performance of 3D AGA Ⅳ in 20 wt% Brine Under Varied Illumination Intensity 39
1.35 Photos of Upper Surface of p-MF 40
1.36 Preparation Process of p-MF 41
1.37 Photos and SEM Images of Pristine MF and p-MF 42
1.38 The Fluidic Transport Property of Pristine MF and p-MF 43
1.39 Photographs of the Absorption of Brine by p-MF 44
1.40 Infrared Radiation Thermal Image of the 3D AGA IV Equipped with p-MF 45
1.41 The Solar Evaporation Performances of 3D AGA IV Equipped with p-MF Compared with Other Solar Steam Generation Devices 46
1.42 The Evaporation Performance of 3D AGA IV Equipped with p-MF During the Continuous Desalination Test 48
1.43 Inner and Outer Surface Photographs of p-MF-equipped 3D AGA IV During the Continuous Desalination Test 49
1.44 Photographs of Precipitated Salt of Brine Solution in p-MF 50
1.45 Weights of Precipitated Salt of Brine Solution in p-MF 51
1.46 Photographs of 3D AGA IV After the Durability Tests 52
1.47 SEM image of p-MF-equipped 3D AGA IV After the Durability Tests 53
1.48 Photographs of Condensation Device for the Evaporated Water 54
1.49 The Concentration of Na^+ and Ions Rejection of Brines Before and After the Desalination Process 55
1.50 The Concentration of Ions and Ions Rejection of the Seawater Before and After the Desalination Process 56

2. Methods 57

2.1 Materials 57

2.2 Fabrication of CGA 57

2.3 Fabrication of 3D CGAs 58

2.4 Fabrication of 3D Assembled CGAs 58

2.5 Construction of 3D Assembled CGA Equipped with p-MF 58

3. Physical Characterizations and Measurement 59

3.1 Transmission Electron Microscopy (TEM) 59

3.2 Atomic Force Microscope (AFM) 59

3.3 Scanning Electron Microscopy (SEM) 59

3.4 Measurements of Nitrogen Adsorption 59

3.5 X-Ray Diffraction (XRD) 60

3.6 Fourier Transform Infrared (FTIR) Spectroscopy 60

3.7 Thermal Gravimetric Analysis (TGA) 60

3.8 Compression Testing 60

3.9 Water Contact Angle Measurement 60

3.10 Measurement of the Solvent Adsorption Capacity 61

3.11 Optical Measurement 61

3.12 Thermal Conductivity Measurement 61

3.13 Evaluation of Solar-driven Steam Generation in Laboratory 61

3.14 Measurement of Ion Concentration 62

4. Supplementary References 63

Movie S1: Compression test (95% strain) of AGA

Movie S2: Adsorption test of LGA for toluene floating on water

Movie S3: Adsorption test of MGA for chloroform sinking below the water

Movie S4: Fast separation of dichloromethane/water with 3D assembled MGA

Movie S5: Fast separation of toluene/water with 3D assembled MGA

Movie S6: The cyclic absorption/combustion of MGA

Movie S7: The cyclic absorption/compression of MGA

1. Supplementary Figures and Tables

1.1 HRTEM Images of ATP, MMT, and LAP

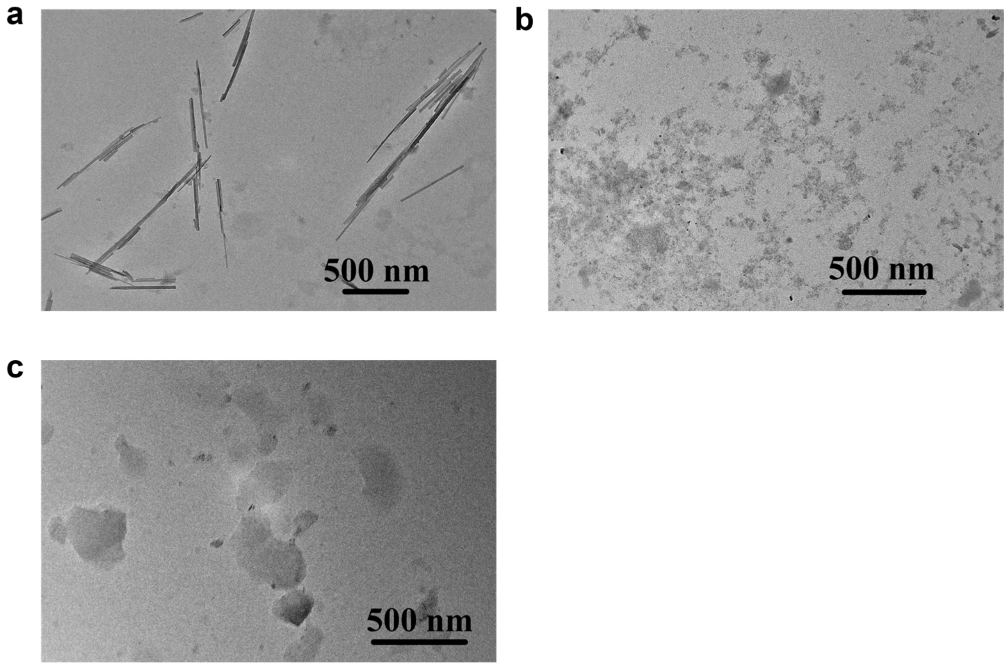

Figure S1. TEM images of (a) ATP, (b) LAP, and (c) MMT.

1.2 Atomic Force Microscopy (AFM) Image of GO

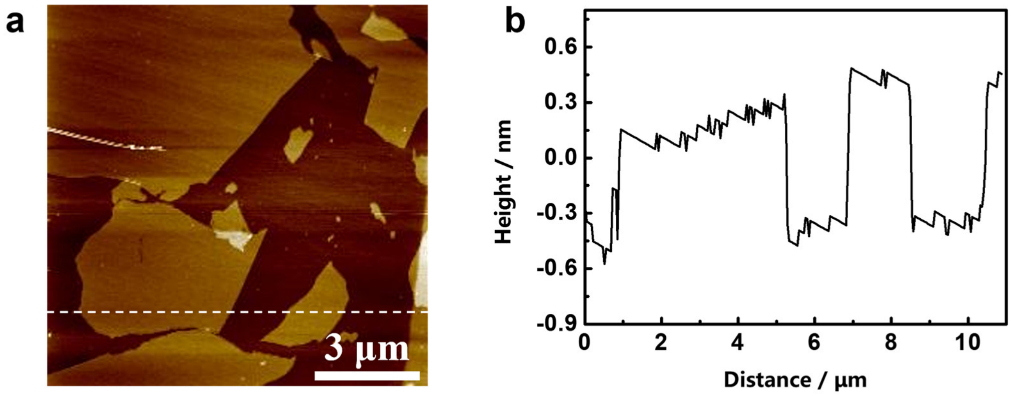

Figure S2. (a) Atomic force microscopy (AFM) image and (b) its corresponding height profile (the white dotted line) show that the graphene oxide (GO) sheets are monolayer (~ 0.9 nm thick).

1.3 SEM Images of MF

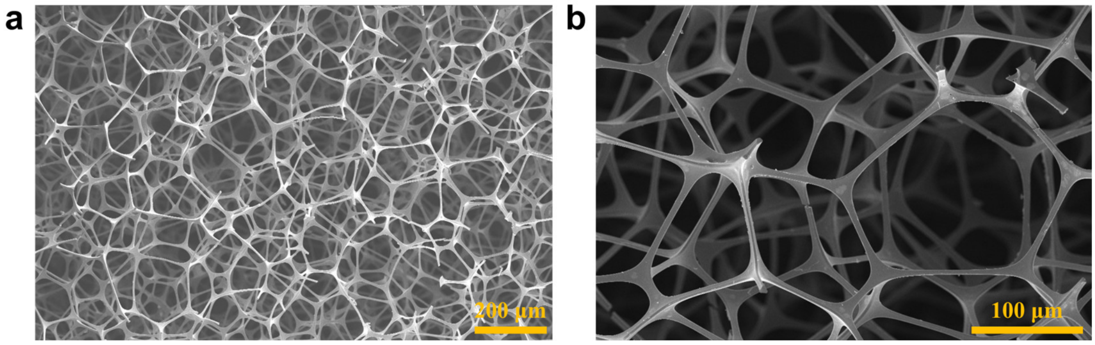

Figure S3. SEM images of MF at (a) low and (b) high magnifications. MF has a 3D interconnected network structure with pores size of ~ 50-150 μm.

1.4 SEM Image of MF/RGO/Clay Composite

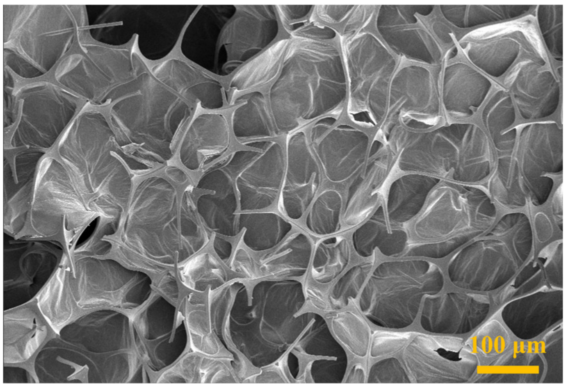

Figure S4. SEM image of clay/RGO/MF composite. RGO sheets self-assembled into network structures in the skeleton of MF, preventing serious stacking of RGO sheets.

1.5 SEM Images of GA Prepared Without Using MF as Skeleton

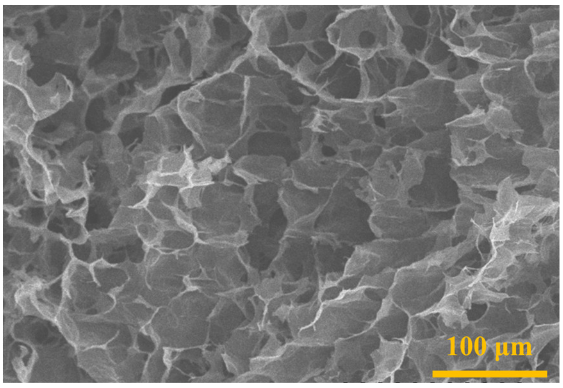

Figure S5. SEM images of GA prepared without using MF as sacrificial skeleton.

1.6 Photos of Clay/GO Suspension and Clay/RGO Hydrogel

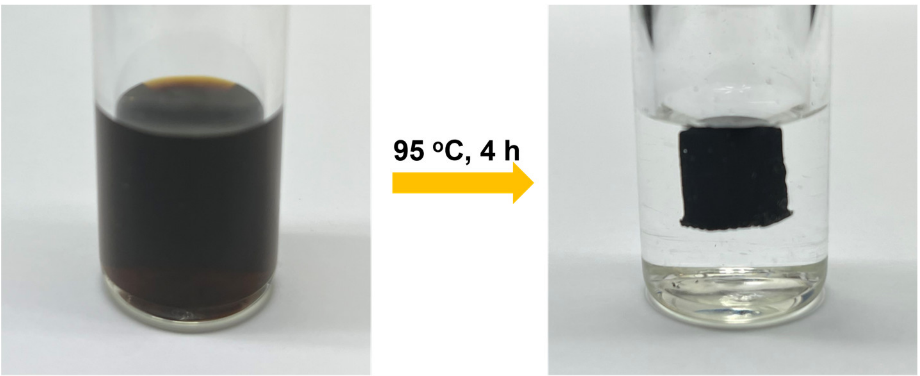

Figure S6. Photos of clay/GO suspension and clay/RGO hydrogel without MF as a sacrificial skeleton. During the reduction process, the enhancement of π-π stacking interactions between RGO sheets, resulting in the volume shrinkage of clay/RGO hydrogel.

1.7 The Densities of CGAs

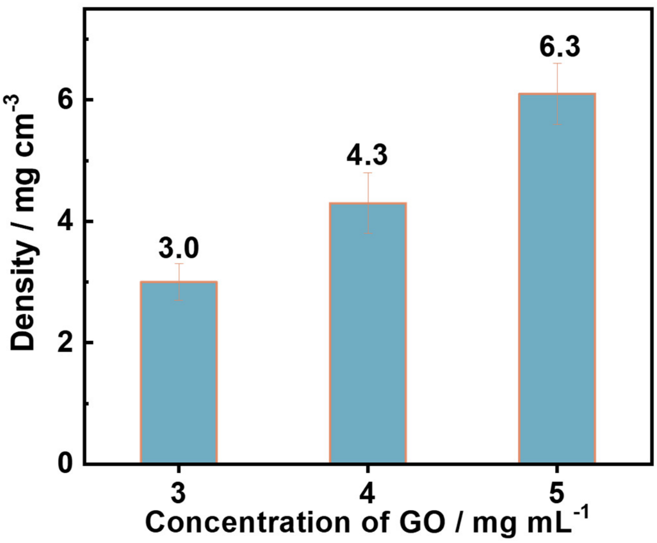

Figure S7. The densities of CGAs as a function of GO concentration.

1.8 Schematic Diagram of Gap Repair During 3D CGA Assembly Process

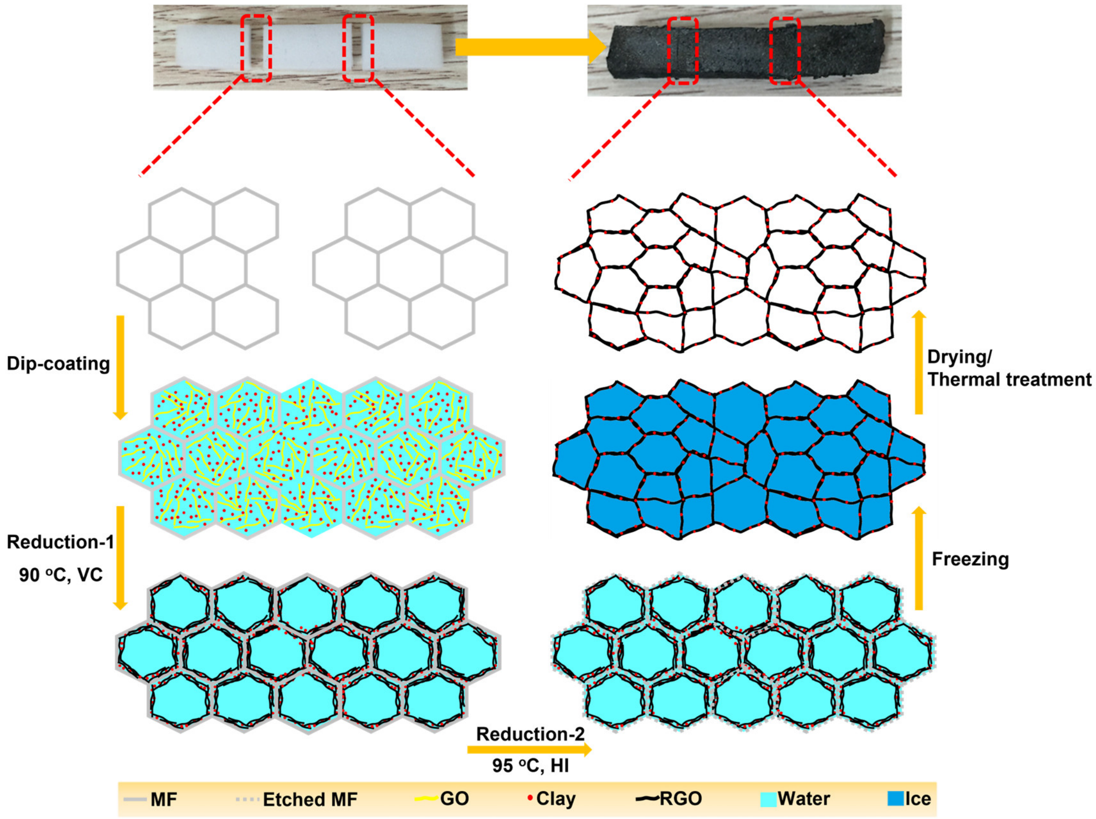

Figure S8. The schematic diagram to describe how the gap are repaired during a 3D CGA assembly process. MFs were immersed into the clay/GO mixed solution, followed by stacking together tightly. During the reduction-1 process, GO sheets reduced to RGO sheets, which self-assembled into RGO network throughout the gaps between adjacent MFs. During the reduction-2 process, the obtained clay/RGO/MF hydrogel was immersed in hydroiodic acid solution (HI) to remove the MF skeleton and further reduce the functional groups on RGO sheets, resulting in the formation of clay/RGO network in the gaps between adjacent MFs. The clay/graphene hydrogel was freeze-dried and thermally treated to obtain an assembled CGA with continuous network.

1.9 SEM Image of the Gap Junction Cross-section of a 3D Assembled CGA

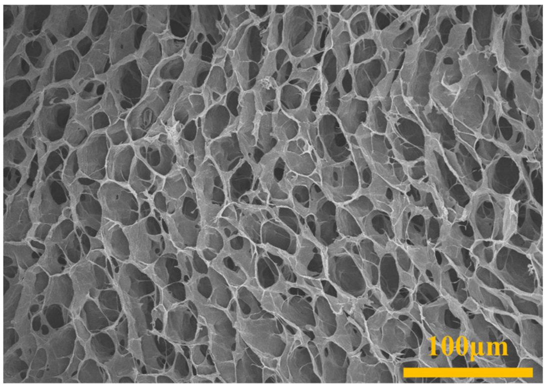

Figure S9. SEM image of gap junction cross-section of a 3D assembled CGA. After assembling into a 3D CGA, the gaps between MFs were completely repaired and presents a unique porous structure.

1.10 SEM Image of GA

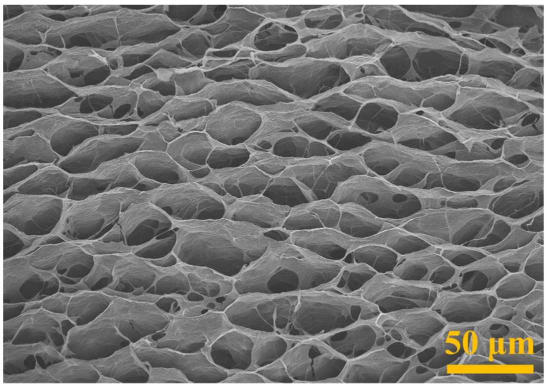

Figure S10. SEM image of GA. As shown in Figure 2a-c, GA shows a similar porous structure to AGA, LGA, and MGA.

1.11 Specific Surface Area of GA and CGAs

Table S1. BET surface area of GA and CGAs.

Sample	GA	AGA	LGA	MGA
BET surface area (m^2 g^{-1})	29	69	59	15

1.12 SEM Image of AGA with Low Magnification

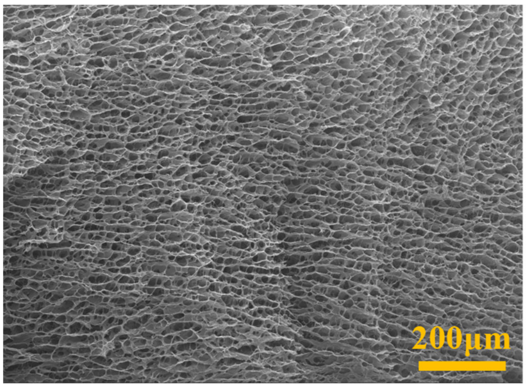

Figure S11. SEM image of AGA with low magnification. AGA shows a large-area (＞1 mm^2) uniform porous structure.

1.13 TEM Images of GO and GA

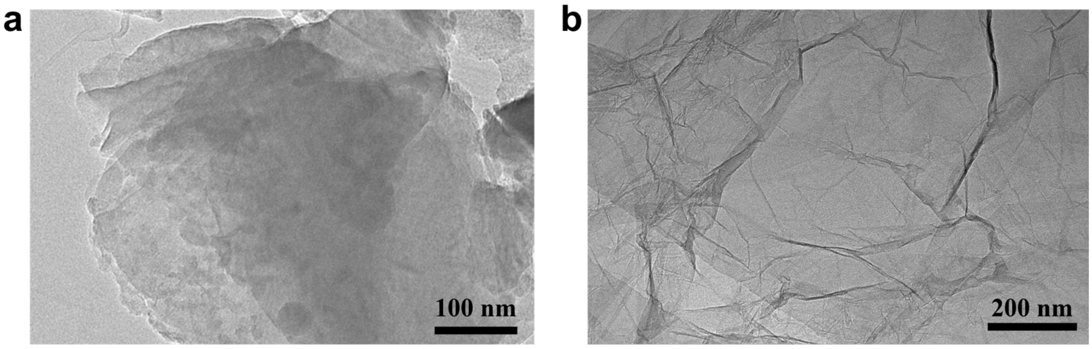

Figure S12. TEM images of GO (a) and GA (b). The smooth surface of the GO sheets formed wrinkle regions after reduction process.

1.14 SEM Elemental Mapping Images of LGA

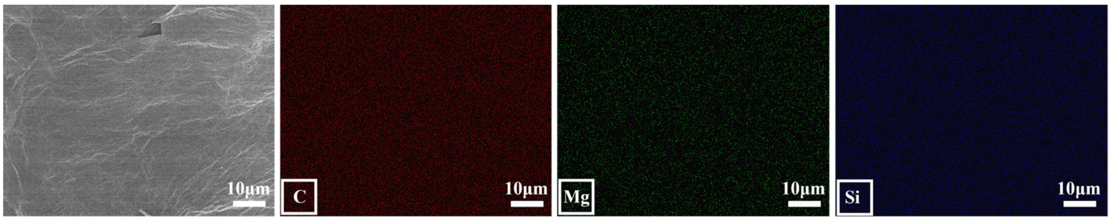

Figure S13. SEM elemental mapping images of LGA. LAP are homogeneously dispersed into the network structure of LGA.

1.15 XRD Patterns of GO and GA

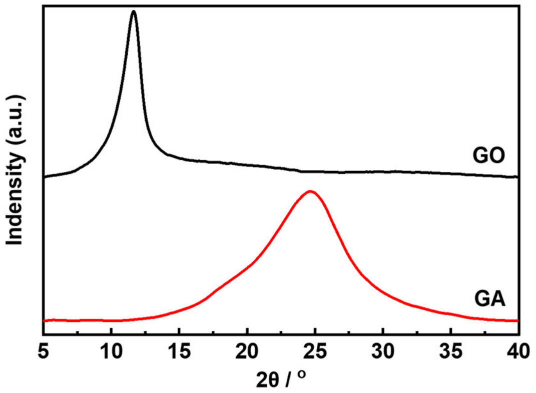

Figure S14. XRD patterns of GO and GA.

1.16 TGA curves of GA and CGAs

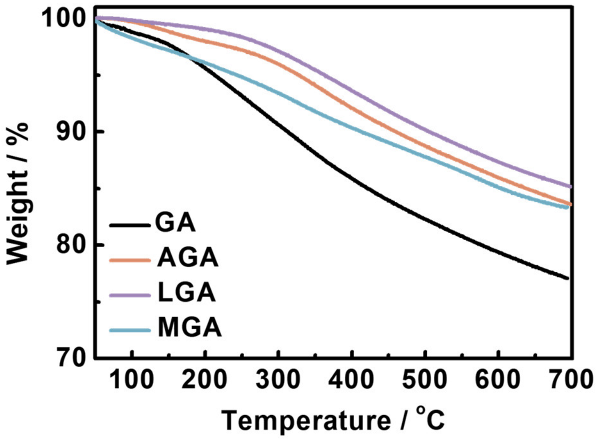

Figure S15. TGA curves of AGA, LGA, MGA, and GA. TGA curves show that the CGA has better thermal stability than GA.

1.17 The Compression Performances of GA and CGAs

Table S2. Compressive stresses of GA and CGAs at 95% strain.

Sample	Density ($mg\ cm^{-3}$)	Compressive stress at 95% strain (MPa)
GA	4.3	0.144
AGA	4.4	0.122
LGA	4.3	0.123
MGA	4.1	0.125

1.18 The Compression Performances of CGAs with Different Densities

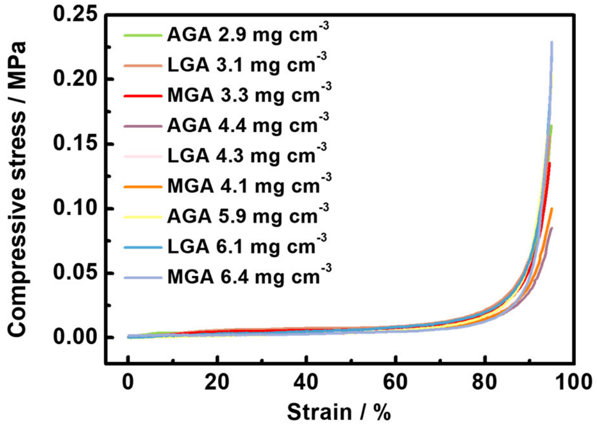

Figure S16. Compressive stresses of CGAs with different densities at 95% strain.

1.19 Comparison of the Compression Performances of CGAs with Other Elastic Aerogels

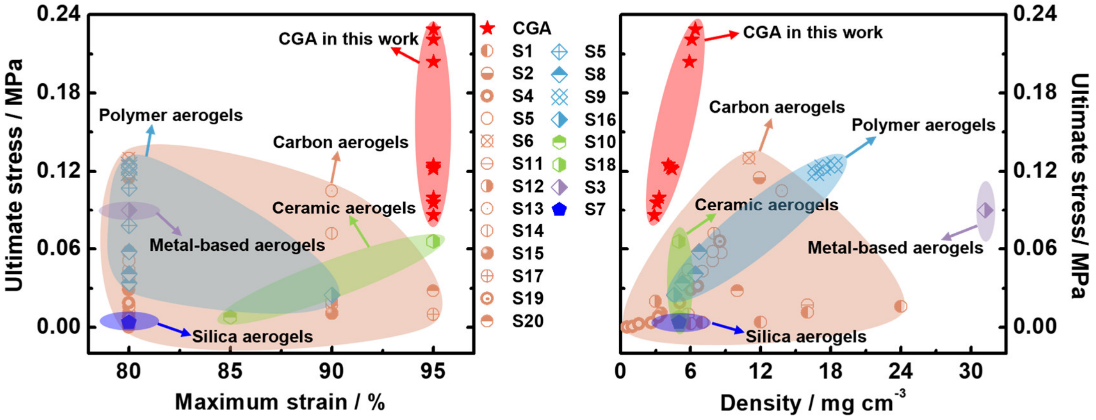

Figure S17. The ultimate stresses of CGAs were compared with those of other elastic aerogels as a function of the maximum strain and density, respectively.

1.20 Comparison of the Compression Performances of CGAs with Other Elastic Porous Materials

Table S3. Compressive properties of CGAs compared to those of other previously reported elastic porous materials.

Sample	Density [mg cm^{-3}]	Maximum strain (%)	Ultimate stress (MPa)	Ultimate stress / Density MPa / mg cm^{-3}	Ref
UCNT aerogel	6	80	0.0031	0.00052	S1
UCNT aerogel	7	80	0.0038	0.00054	S1
UCNT aerogel	12	80	0.004	0.00033	S1
UCNT aerogel	16	80	0.0115	0.000722	S1
UCNT aerogel	24	80	0.016	0.00066	S1
CNT sponge	11.88	80	0.115	0.00968	S2
RF-GO-metal compressible aerogel	31.2	80	0.09	0.00288	S3
Graphene monolith	0.56	80	0.00031	0.00055	S4
Graphene monolith	0.99	80	0.00084	0.00085	S4
Graphene monolith	1.55	80	0.00271	0.00175	S4
Graphene monolith	2.65	80	0.00361	0.00136	S4
Graphene monolith	3.25	80	0.00792	0.00244	S4
Graphene monolith	5.10	80	0.019	0.00373	S4
Graphene monolith	6.00	80	0.029	0.00483	S4
Graphene monolith	6.62	80	0.032	0.00483	S4
G1P0.5-f	5.5	80	0.038	0.00691	S5
G1P0.5-v	7.0	80	0.043	0.00614	S5
G1P0.5-a	8.6	80	0.057	0.00663	S5
G1P1-f	5.8	80	0.044	0.00759	S5
G1P1-v	7.9	80	0.051	0.00646	S5
G1Pa-a	7.9	80	0.058	0.00734	S5
rGO-porous network	11	80	~0.13	0.01182	S6
SiO_2 aerogel	5.0	80	0.004	0.0008	S7

PAA(20%)/graphene aerogel	5.3	80	0.034	0.00642	S8
PAA(30%)/graphene aerogel	6.4	80	0.041	0.00641	S8
PAA(50%)/graphene aerogel	6.7	80	0.058	0.00866	S8
PDMS/graphene aerogel	72	80	0.107	0.00149	S5
TPI/ graphene aerogel	56	80	0.078	0.00139	S5
GCLN-5	16.7	80	0.119	0.00713	S9
GCLN-10	17.3	80	0.123	0.00711	S9
GCLN-20	18.3	80	0.125	0.00683	S9
Silica nanofibrous aerogel	5	85	0.008	0.0016	S10
Graphene aerogels	~16	90	0.017	0.00106	S11
Ultralight graphene aerogels (ULGAs)	~3	90	0.02	0.00667	S12
C-G Monolith	13.8	90	0.105	0.00761	S13
Graphene / CNTs hybird foams	~8	90	~0.072	0.009	S14
HPF GA	3.5	90	0.011	0.00314	S15
GA/NRL-0.5	4.6	90	0.025	0.0054	S16
Fe_3O_4/graphene aerogels	5.8	95	0.01	0.00172	S17
hBNAGs ceramic aerogel	5	95	~0.066	0.0132	S18
BHGM	8.5	95	0.066	0.00776	S19
Graphene aerogels	10	95	0.028	0.0028	S20
AGA	2.9	95	0.086	0.02976	This work
LGA	3.1	95	0.096	0.03094	This work
MGA	3.3	95	0.100	0.03021	This work
AGA	4.4	95	0.122	0.02773	This work
LGA	4.3	95	0.123	0.0286	This work
MGA	4.1	95	0.125	0.03049	This work
AGA	5.9	95	0.204	0.03466	This work
LGA	6.1	95	0.221	0.03616	This work
MGA	6.4	95	0.229	0.03584	This work

1.21 SEM Image of AGA After a Series of Harsh Tests

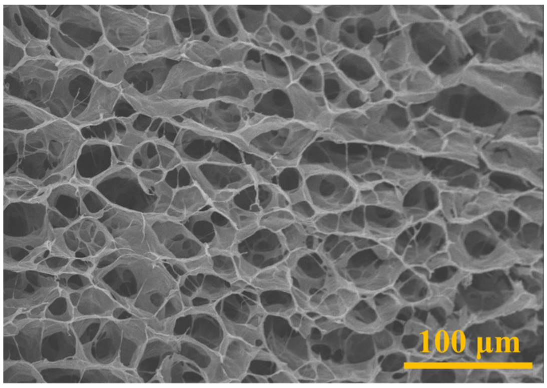

Figure S18. SEM image of AGA after a series of harsh tests.

1.22 Fast Separation of Organic Solvent/Water with 3D Assembled MGA

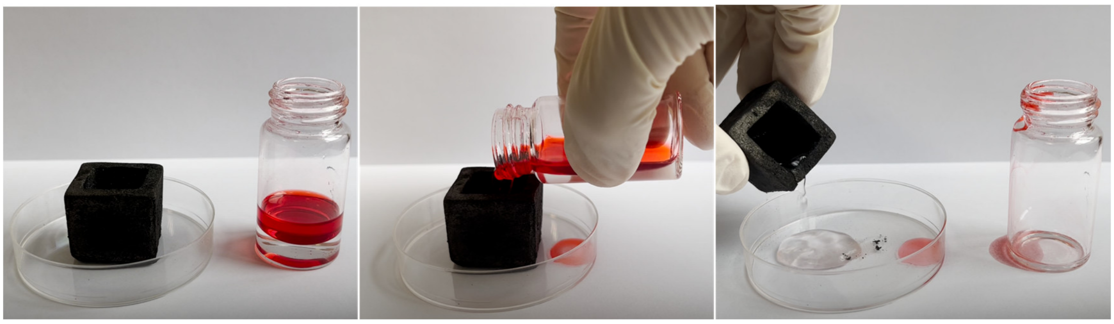

Figure S19. Fast separation of toluene (dyed with Sudan III) from water with the 3D cubic cup-shaped MGA with within several seconds. When the toluene (dyed with Sudan III)/water mixture was poured into the cubic cup-shaped MGA, the toluene was rapidly absorbed, resulting in a complete separation of water and toluene within several seconds.

1.23 Adsorption Capacities of the GA

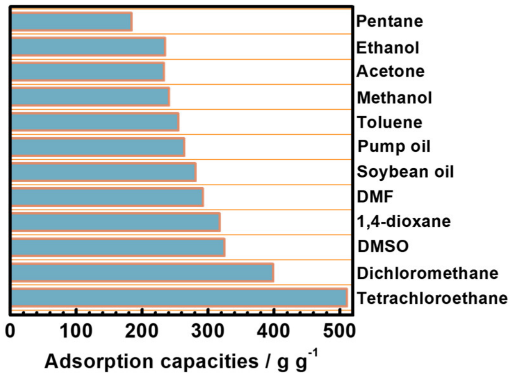

Figure S20. Adsorption capacities of the GA measured for a range of organic solvents in terms of their densities.

1.24 Adsorption Capacities of the LGA, MGA, and GA

Table S4. Adsorption capacities of the LGA, MGA, and GA measured for various organic solvents in terms of their densities.

Organics	Density [g mL^{-1}]	LGA [g g^{-1}]	MGA [g g^{-1}]	GA [g g^{-1}]
Pentane	0.626	186	190	184
Ethanol	0.789	226	233	235
Acetone	0.79	237	246	233
Methanol	0.79	231	238	241
Toluene	0.87	262	267	255
Pump oil	0.88	267	266	264
Soybean oil	0.92	277	274	281
DMF	0.95	279	284	292
1,4-dioxane	1.03	310	324	318
DMSO	1.10	322	335	325
Dichloromethane	1.33	407	415	399
Tetrachloroethane	1.56	495	519	511

1.25 Comparison of Adsorption Capacities of CGAs and other Porous Materials Reported in the Literatures

Table S5. Comparison of adsorption capacities of CGAs and other porous materials measured for various organic solvents.

Materials	Solvents	Q [g g^{-1}]	Ref
CNT foam	Ethanol, ethylene glycol, hexane, chloroform	80~180	S21
Graphene/α-FeOOH	Cyclohexane, toluene, phenoxin	13~27	S22
Spongy graphene	DMSO, THF, acetone, methanol, ethanol, chloroform, hexane, heptane, octane, decane, dodecane, toluene, nitrobenzene, 1,2-dichlorobenzene, ethylbenzene	20~86	S23
Carbonaceous nanofiber aerogel	Cyclohexane, ethanol, chlorobenzene, phenoxin	40~118	S24
Functionalized graphene aerogel	Chlorobenzene, chloroform, DMF, Toluene, THF, Methanol, Ethanol, Acetone	34~112	S25
Graphene foams	DMF, THF, DMSO, acetone, methanol, ethanol, ethylene glycol, glycerol, dichloromethane, chloroform, 1,2-dichloroethane, tetrachloroethane, cyclohexane, heptane, benzene, toluene, xylene	47-110	S26
Graphene aerogel	n-hexane, n-dodecane, ethanol, tributyl phosphate, n-methyl pyrrolidone, ethylene glycol, dichloromethane, carbon tetrachloride	120~250	S27
Bridged silsesquioxane aerogel	Ethanol, isopropanol, ethylene glycol, acetone, n-hexane, heptane, cyclohexane, chloroform, carbon tetrachloride, ethyl acetate, methacrylic acid, tetrahydrofuran, dimethyl sulfoxide, toluene, styrene	12~29	S28
Silicone sponge	Sunflower oil, gasoline, diesel, crude oil, petroleum ether, n-hexadecane, oleic acid, n-octane, n-hexane, o-dichlorobenzene, toluene, dichloromethane, acetone	6~14	S29
Carbon foam	THF, ethanol, ethylene glycol, phenoxin, chloroform, h-hexane, toluene, chlorobenzene, petroleum ether, ethyl acetate, lactic acid	18~48	S30
PU foam	Octane, decane, dodecane	18~25	S31
Carbon Fiber Aerogel	Cyclohexane, ethanol, acetone, DMF, benzyalcohol, hexane, heptane, isopropyl alcohol, octadecylene, chloroform	5~12	S32
Nanocellulosic hybrid aerogel	Chloroform, tetrachloroethane, methylbenzene, n-hexane, petroleum ether, diethyl ether, acetone, methyl alcohol, n-octane, diesel oil, pump oil, soybean oil, silicone oil.	93-203	S33
Graphene aerogel	n-hexane, acetone, ethanol, toluene, 1,4-dioxane, ethylene glycol, chloroform, tetrachloroethane	176-513	S34
PVDF/SiO_2@GO nanofibrous aerogel	n-Hexane, kerosene, toluene, cooking oil, dichloromethane, chloroform	129-264	S35
COF/graphene aerogel	Dimethyl sulfoxide, dimethylformamide, dimethylacetamide, dioxane, tetrahydrofuran, phenoxin, silicone oil, chloroform, ethylene glycol, toluene, acetone, ethanol, ethyl acetate, hexane, methanol, cyclohexane	98-240	S36
LGA and MGA	Pentane, ethanol, acetone, methanol, toluene, pump oil, soybean oil, dmf, 1,4-dioxane, dmso, dichloromethane, tetrachloroethane	186~519	This Work

1.26 Schematic Illustration for The Experimental Setup of The Solar Steam Generation Test

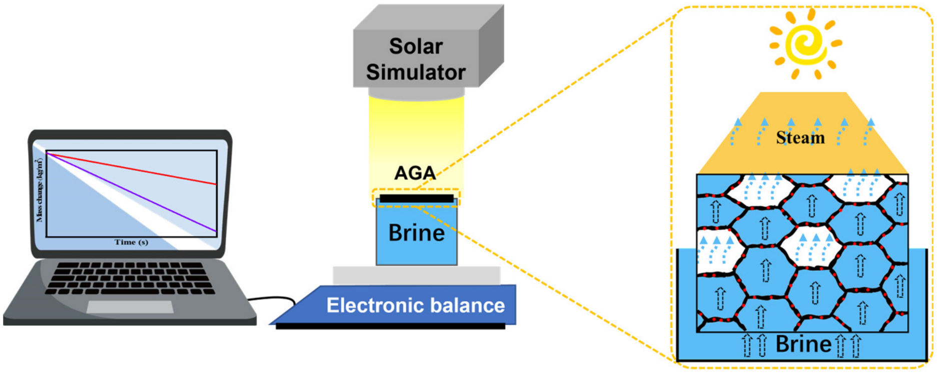

Figure S21. Schematic illustration for the experimental setup of the solar steam generation test of AGA.

1.27 The Consturction of 3D AGA I

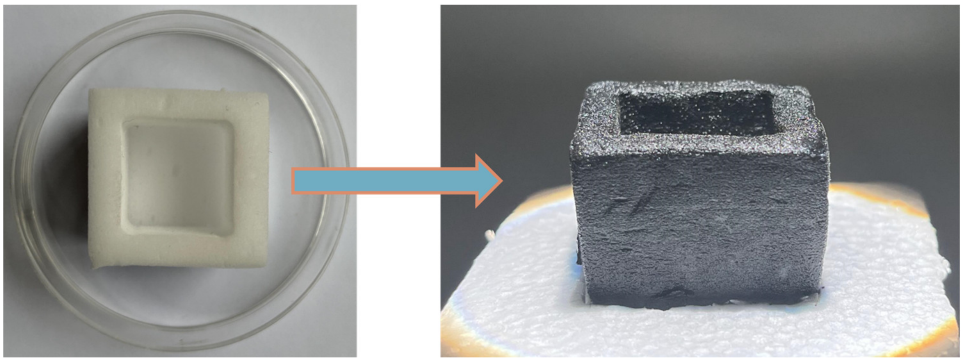

Figure S22. Based on the skeleton of 3D MF, a 3D AGA I (30 mm × 30 mm × 20 mm) was fabricated.

1.28 Continuous Desalination Test for 2D AGA

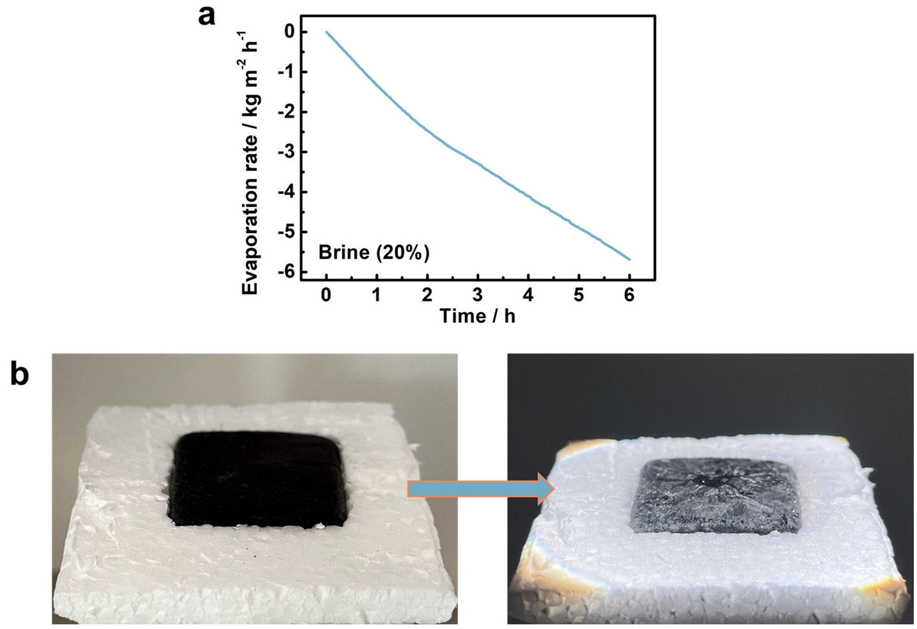

Figure S23. (a) Mass change of brine (20 wt%) for 6 h under one sun illumination. After ~1.5 h of continuous desalination, the evaporation rate gradually decreased. (b) Salt accumulation appeared on the surface of 2D AGA during continuous solar desalination.

1.29 Continuous Desalination Test for 3D AGA II

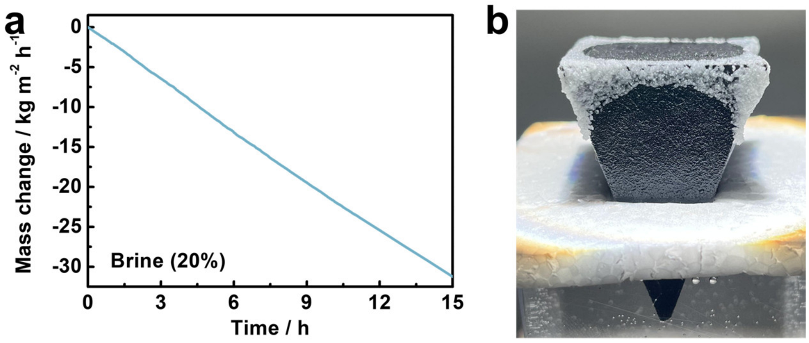

Figure S24. (a) Mass change of brine (20 wt%) for 15 h under one sun illumination; The average evaporation rates for continuous desalination of 15 h can be maintained at ~2.00 kg m^{-2} h^{-1}; (b) During continuous 15 h desalination process, the salt crystallized at the edge of the 3D AGA II.

1.30 Continuous Desalination Test for the 3D AGA III

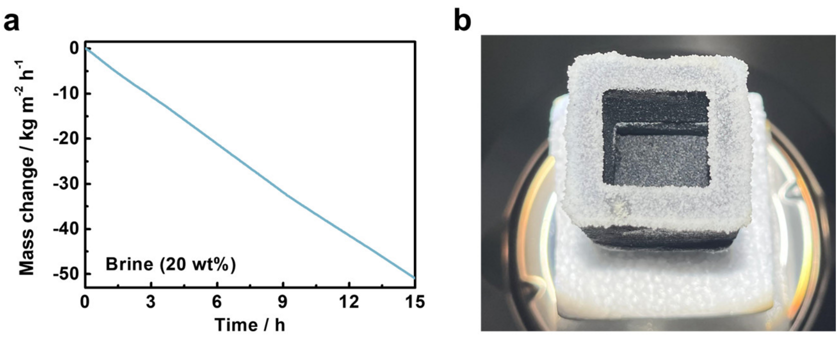

Figure S25. (a) Mass change of brine (20 wt%) for 15 h under one sun illumination. The average evaporation rate of the 3D AGA III is ~3.40 kg m^{-2} h^{-1}; (b) As the desalination experiment proceeds, salt accumulation occurred on the upper surface of 3D AGA III, while no salt deposition occurred on the bottom and walls of cubic-cup part.

1.31 Schematic Illustration of the Continuous Desalination Test for the 3D AGA III Under 1 and 3 Sun Illumination

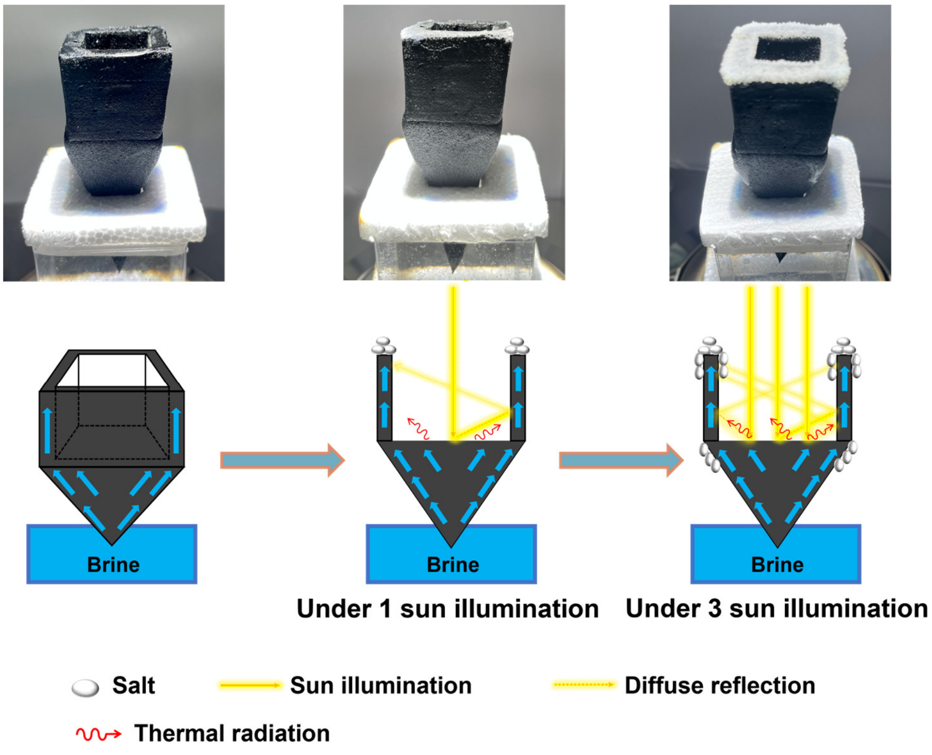

Figure S26. Schematic illustration of the continuous desalination test for the 3D AGA III under 1 and 3 sun illumination. Due to the cubic-cup part of the 3D AGA III was the same in cross-section perpendicular to brine flow, the brine flowed along the cubic-cup walls. Under one sun illumination, salt accumulation occurred on the upper surface of the 3D AGA III. With the enhancement of solar-driven evaporation, salt gradually accumulated on the cup walls of the 3D AGA III under 3 sun illumination.

1.32 The Consturction of the 3D AGA IV

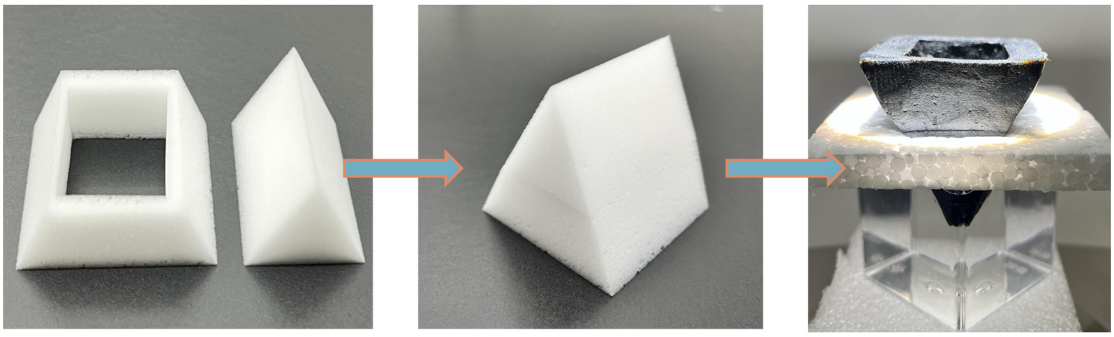

Figure S27. The 3D AGA IV (40 mm × 40 mm × 40 mm) with the expanding cross-section was fabricated.

1.33 Schematic Illustration of the Continuous Desalination Test for the 3D AGA IV Under 3 Sun Illumination

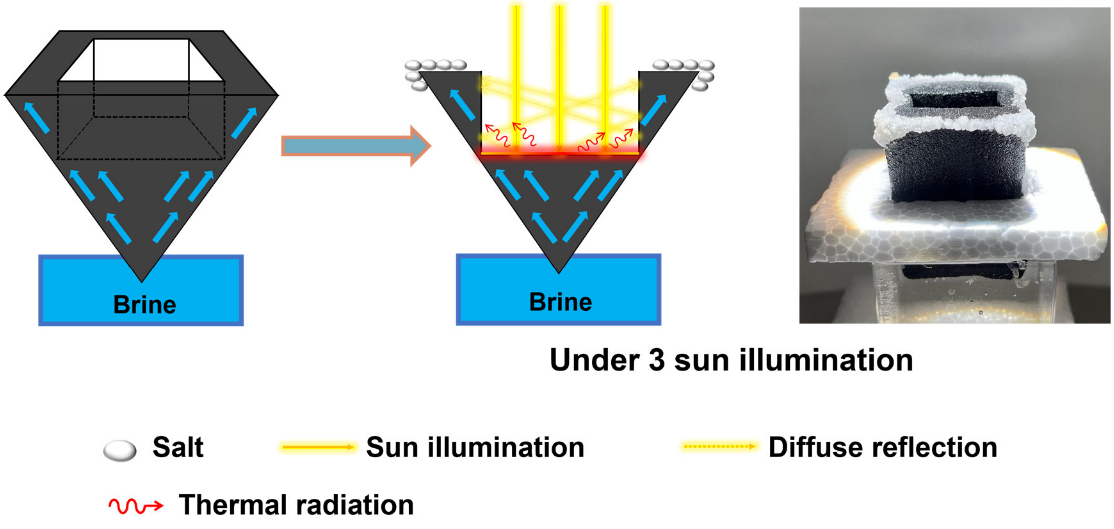

Figure S28. Schematic illustration of the continuous desalination test for the 3D AGA IV under 3 sun illumination. Due to the cup part of the 3D AGA IV had the expanding cross-section perpendicular to brine flow, the brine flowed along the cubic-cup walls. The edge-preferential salt crystallization is achieved by the effect of the inhomogeneous brine transport along the radial direction.

1.34 The Evaporation Performance of 3D AGA IV in 20 wt% Brine Under Varied Illumination Intensity

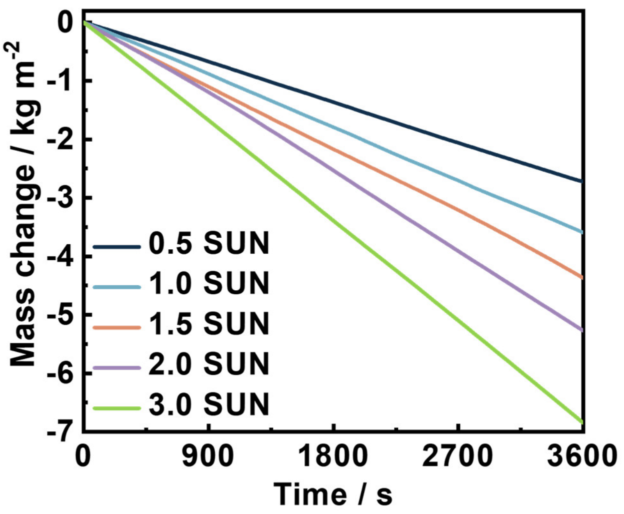

Figure S29. The mass change of water versus time with 3D AGA IV in 20 wt% brine under varied illumination intensity.

1.35 Photos of Upper Surface of p-MF

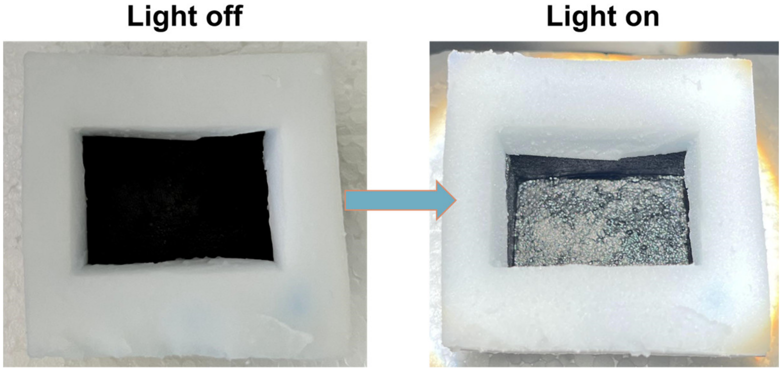

Figure S30. The p-MF has a hole with the same size as the inner diameter of the 3D AGA IV to allow sunlight to pass through.

1.36 Preparation Process of p-MF

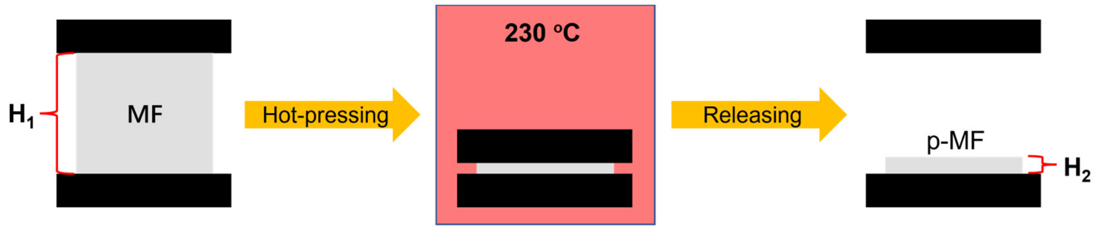

Figure S31. Schematic of preparation process of p-MF. Pristine MF (H_1: 20 cm) was pressed under 230 °C for 15min to obtain p-MF (H_2: 2 cm).

1.37 Photos and SEM Images of Pristine MF and p-MF

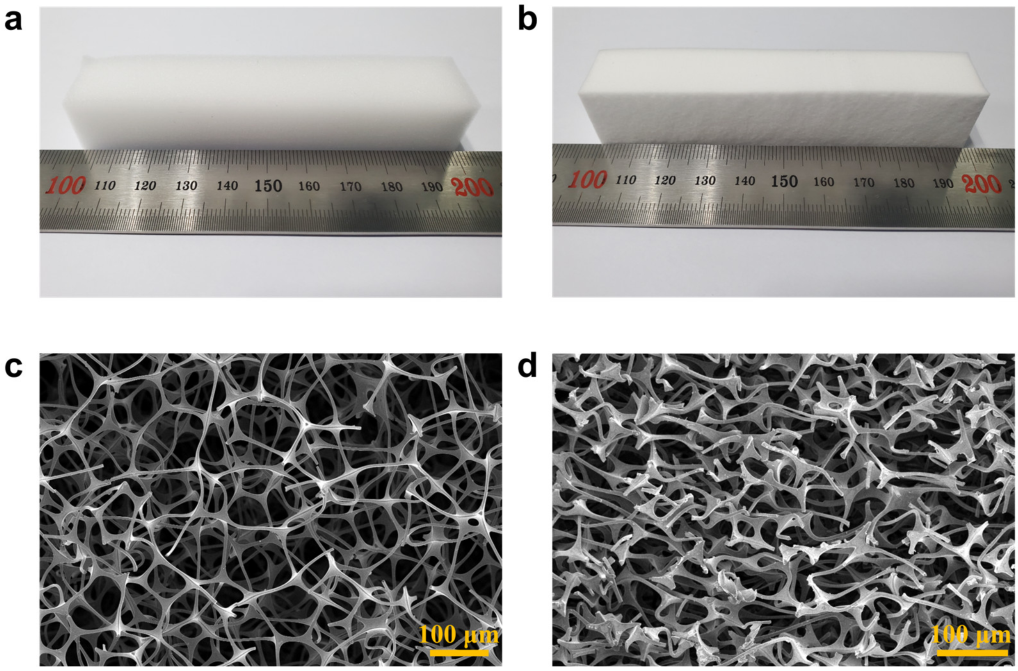

Figure S32. Photographs of (a) pristine MF and (b) p-MF. SEM images of (c) pristine MF and (d) p-MF.

1.38 The Fluidic Transport Property of Pristine MF and p-MF

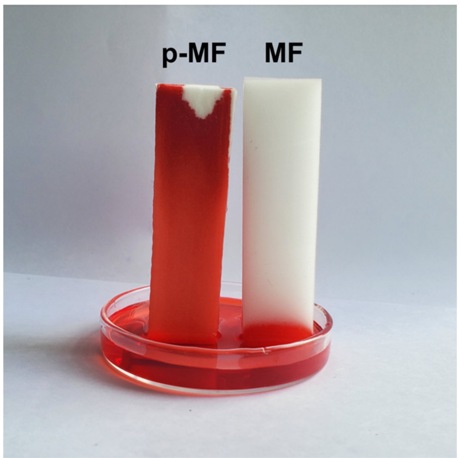

Figure S33. The photograph of pristine MF and p-MF after red ink adsorption. P-MF showed better fluidic transport property than pristine MF.

1.39 Photographs of the Absorption of Brine by p-MF

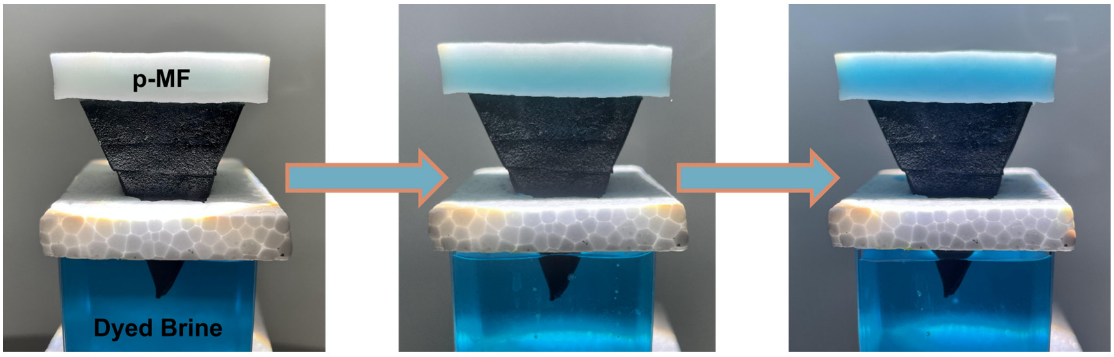

Figure S34. Photographs of the absorption of brine by p-MF. The flexuous network constructed by p-MF can absorb salt driven by the salt concentration gradient. To get better visual contrast, the brine was dyed by copper sulfate.

1.40 Infrared Radiation Thermal Image of the 3D AGA IV Equipped with p-MF

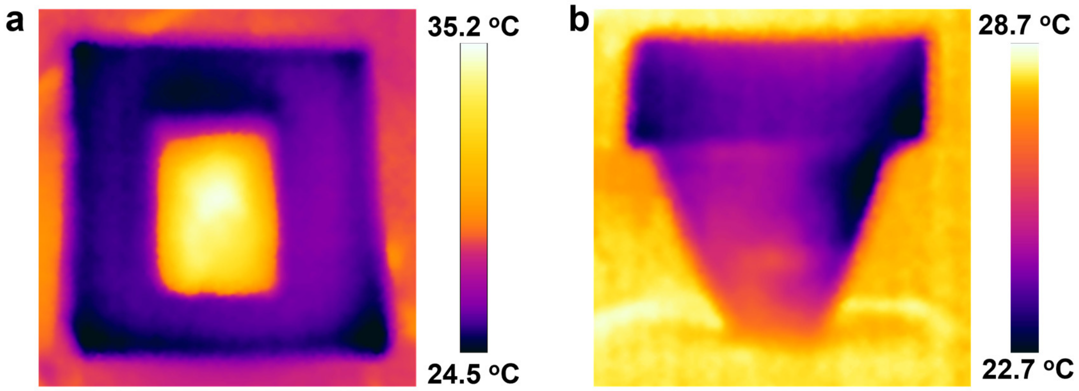

Figure S35. Infrared radiation thermal images of the (a) upper surface and (b) side surface of 3D AGA IV equipped with p-MF under one sun illumination. The temperature on the side surface of the 3D AGA IV is lower than the ambient temperature. Therefore, the 3D AGA IV can harvest energy from the environment to enhance evaporation performance.

1.41 The Solar Evaporation Performances of 3D AGA IV Equipped with p-MF Compared with Other Solar Steam Generation Devices

Table S6. The evaporation rates of 3D AGA IV equipped with p-MF under a wide salinity brine (0% ~ 25%) compared with those of other previously reported solar steam generation devices.

Sample	Salinity (%)	Water evaporation rate (kg m^{-2} h^{-1})	Optical concentration (kW m^{-2})	Ref
3D SCS cup	0	1.7	1	S37
3D SCS cup	3.5	1.65	1	S37
3D SCS cup	10	1.54	1	S37
3D SCS cup	15	1.46	1	S37
3D SCS cup	20	1.4	1	S37
3D SCS cup	25	1.36	1	S37
Sunflower pith with hydrogel coating	0	1.9	1	S38
Photothermal roll	0	1.93	1	S39
Photothermal roll	3.5	1.68	1	S39
3D a-MoC1x@CB cones	20	2.8	1	S40
Self-regenerating evaporator	3.5	1.46	1	S41
Self-regenerating evaporator	20	1.04	1	S41
Janus absorber	15	0.8	1	S42
Janus-interface solar-steam generator	0	2.21	1	S43
MoS_2/SA@MF hybrid sponge	3.5	1.92	1	S44
MoS_2/SA@MF hybrid sponge	25	1.78	1	S44
Janus evaporator	3.5	1.38	1	S45
Janus evaporator	10	1.35	1	S45
Solar steam generator	3.5	1.42	1	S46
T-shaped synthetic tree	3.5	2.03	1	S47
Bilayer polymer foam	0	1.57	1	S48
Bilayer polymer foam	3.5	1.59	1	S48
30° cone-shaped Janus evaporator	3.5	1.73	1	S49
Lily-inspired hierarchical structure	10	1.2	1	S50
VSI-700	3.0	1.64	1	S51

VSI-700	3.5	1.63	1	S51
VSI-700	7.5	1.61	1	S51
VSI-700	15	1.59	1	S51
Superhydrophilic porous carbon foam	0	1.57	1	S52
Janus absorber	3.5	1.13	1	S53
Vasculatural PVA/PB hydrogel	3.5	3.44	1	S54
Biomimetic 3D evaporator	25	2.63	1	S55
Conical frustum evaporator	3.5	2.26	1	S56
Conical frustum evaporator	10	2.23	1	S56
Conical frustum evaporator	15	2.15	1	S56
Conical frustum evaporator	20	1.95	1	S56
Conical frustum evaporator	25	1.95	1	S56
PDA/HBC on V-85	3.5	1.6	1	S57
PDA/HBC on V-85	20	1.35	1	S57
PDA/HBC on V-85	25	1.25	1	S57
Hanging PANi-cotton fabric	3.5	1.94	1	S58
Sponge-M-BHNPs	0	3.2	1	S59
Sponge-M-BHNPs	3.5	2.9	1	S59
Dual-zone photothermal sphere	3.5	2.6	1	S60
Dual-zone photothermal sphere	7	2.3	1	S60
Dual-zone photothermal sphere	20	2.1	1	S60
Carbonized corncob	0	4.16	1	S61
Carbonized corncob	3.5	3.74	1	S61
RGO bundles	0	3	1	S62
Polyzwitterionic hydrogel	10	4.14	1	S63
Thermoelectricity-freshwater cogenerator	0	1.79	1	S64
3D AGA IV equipped with p-MF	3.5	4.45	1	This work
3D AGA IV equipped with p-MF	10	4.34	1	This work
3D AGA IV equipped with p-MF	15	4.25	1	This work
3D AGA IV equipped with p-MF	20	4.13	1	This work
3D AGA IV equipped with p-MF	25	3.98	1	This work

1.42 The Evaporation Performance of 3D AGA IV Equipped with p-MF During the Continuous Desalination Test

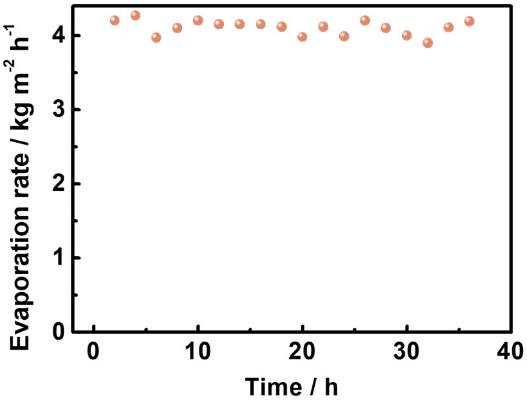

Figure S36. Mass change of brine (20 wt%) for continuous 36 h illumination (one sun). 3D AGA IV equipped with p-MF showed a high average evaporation rate of ~4.10 kg m^{-2} h^{-1}.

1.43 Inner and Outer Surface Photographs of p-MF-equipped 3D AGA IV During the Continuous Desalination Test

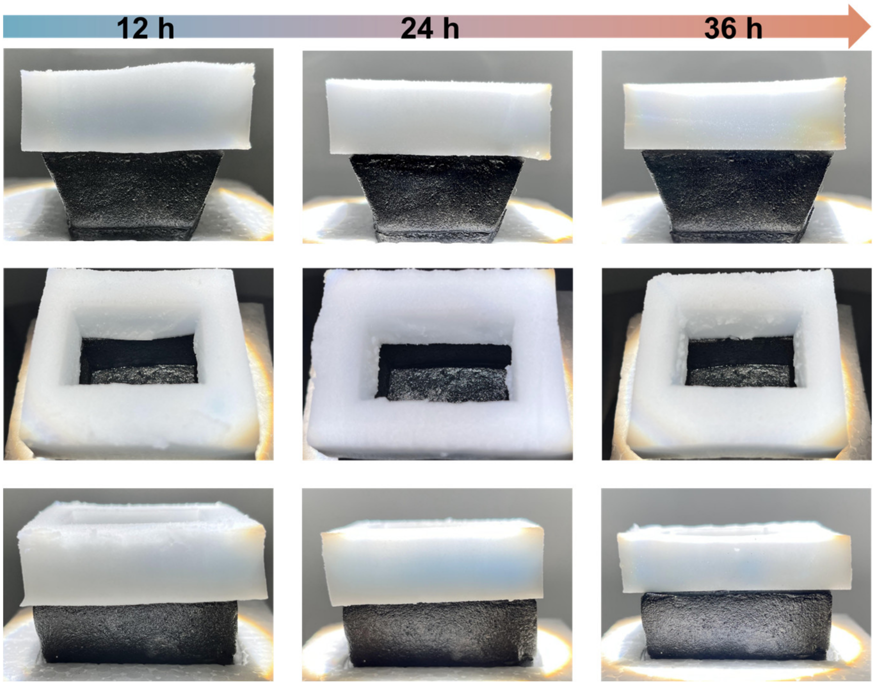

Figure S37. Photographs of the p-MF-equipped 3D AGA IV at 12, 24, and 36 h during the continuous desalination test under 1-sun illumination, respectively. No salt accumulation was observed on the inner and outer surface of the 3D AGA IV during continuous desalination process.

1.44 Photographs of Precipitated Salt of Brine Solution in p-MF

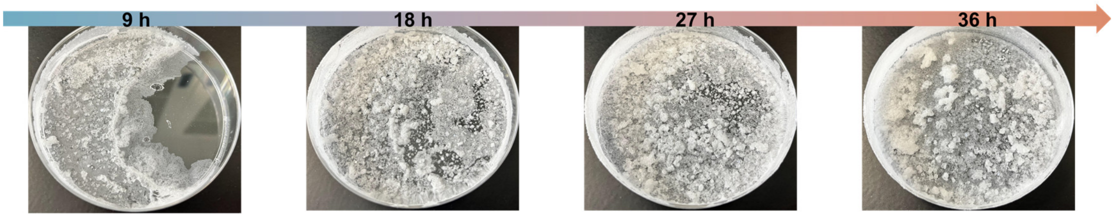

Figure S38. Photographs of precipitated salt collected by drying the brine solution in p-MF every 9 h.

1.45 Weights of Precipitated Salt of Brine Solution in p-MF

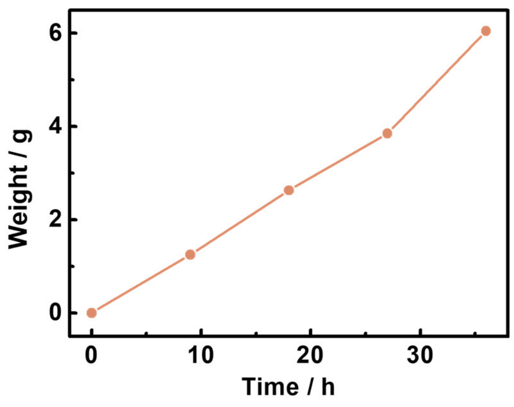

Figure S39. Weights of precipitated salt collected by drying the brine solution in p-MF every 9 h.

1.46 Photographs of 3D AGA IV After the Durability Tests

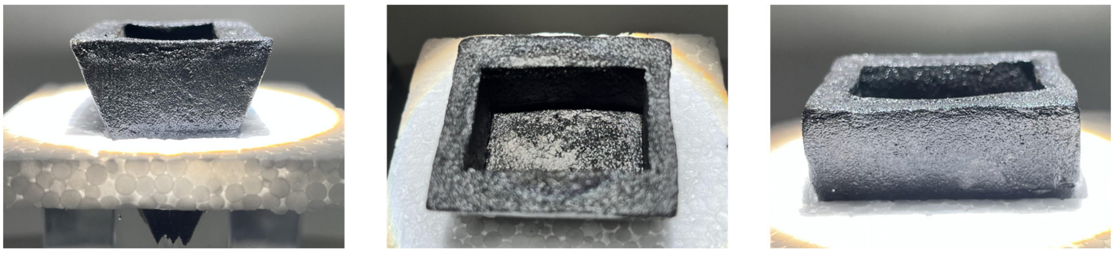

Figure S40. The p-MF-equipped 3D AGA IV was floating on the surface of highly concentrated solutions (20 wt% NaCl) for 7 days and irradiated under one sun irradiation for 18 h continuous desalination per day. No salt accumulation was observed on the inner and outer surface of the 3D AGA IV after the durability tests.

1.47 SEM image of p-MF-equipped 3D AGA IV After the Durability Tests

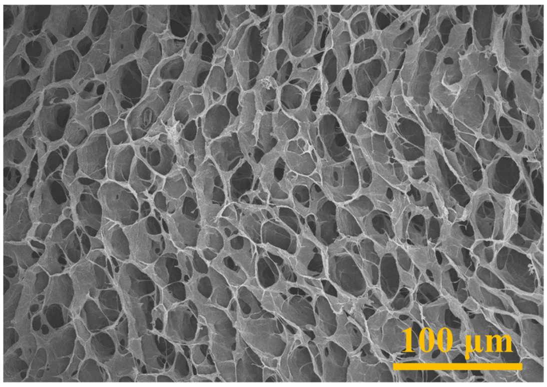

Figure S41. SEM image of the p-MF-equipped 3D AGA IV after the durability tests (Floating for 7 days, 18 h continuous desalination under one sun irradiation per day). The appearances (Figure S40) and microstructures of 3D AGA IV before and after durability tests remained unchanged, indicating the durability and excellent structural stability.

1.48 Photographs of Condensation Device for the Evaporated Water

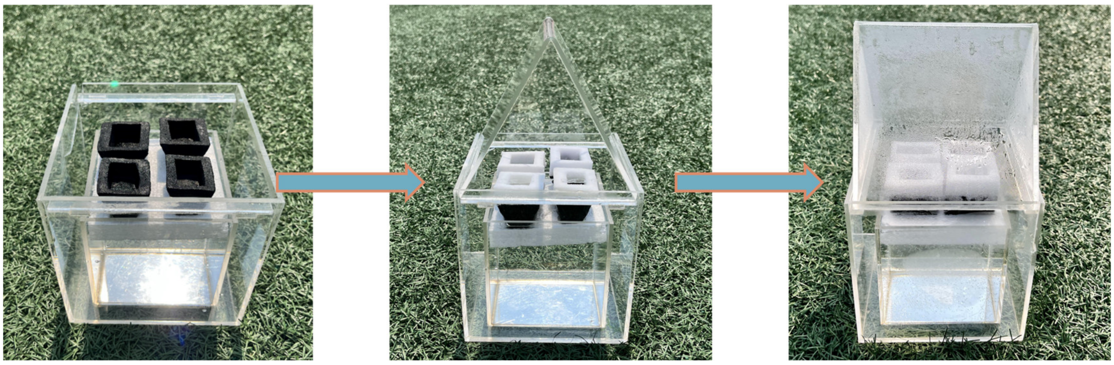

Figure S42. Photographs of the designed solar desalination device based on p-MF-equipped 3D AGA IV.

1.49 The Concentration of Na^+ and Ions Rejection of Brines Before and After the Desalination Process

Table S7. The concentration (mg L^{-1}) of Na^+ of a wide salinity brine before and after the desalination process.

Salinity [%]	Before [mg L^{-1}]	After [mg L^{-1}]	Rejection [%]
3.5	35000	3.5	99.99
10	100000	5.6	99.99
15	150000	6.5	99.99
20	200000	6.8	99.99
25	250000	7.3	99.99

1.50 The Concentration of Ions and Ions Rejection of the Seawater Before and After the Desalination Process

Table S8. The concentration (mg L^{-1}) of ions of the seawater (from Hongdao, Qingdao, China) before and after the desalination process.

Ion	Before [mg L^{-1}]	After [mg L^{-1}]	Rejection [%]
Na^{+}	9200	0.42	99.99
Mg^{2+}	856	0.13	99.99
K^{+}	490	0.21	99.99
Ca^{2+}	247	0.24	99.99

2. Methods

2.1 Materials

Graphite powder was obtained from Qingdao Huagao Co., Ltd. (China). LAP was purchased from Rockwood Company (USA). MMT was purchased from Sigma-Aldrich Pty., Ltd. (USA). ATP was purchased from ITC Company (USA). Ascorbic acid and hydroiodic acid were purchased from Sinopharm Chemical Reagent Co., Ltd. (China). $KMnO_4$, concentrated H_2SO_4 (98%), concentrated hydrochloric acid (36-38%), and hydrogen peroxide (30%) were all purchased from Beijing Chemical Factory (China). The melamine foam (MF) was provided by Beiyou Company (Shanghai). A dialysis bag was purchased from Beijing Jingke Hongda Biotechnology Co., Ltd. (China). The GO aqueous dispersion was fabricated following our previously reported method.

2.2 Fabrication of CGA

ATP, MMT, and LAP could be dispersed in water to form a stable aqueous suspension. The clay (ATP, LAP, and MMT) suspensions and GO suspension were mixed to obtain uniform mixed suspensions, respectively. Designated amounts of reducing agent (ascorbic acid) were added to clay/GO solutions under vigorous stirring. The weight ratio of GO and ascorbic acid was 1:2, and the concentration of GO solution was 3-5 mg mL^{-1}. MF was cut into the arbitrary-shaped shape as required, and then was immersed into the mixed solution by a squeezing procedure for several times. The composite was heated at 90 °C for 4 h to obtain reduced clay/RGO/MF hydrogel. The resultant hydrogel was put in hydroiodic acid solution (HI, 37%) (the weight ratio of GO and HI was 1:3) at 95 °C for 4 h to remove the MF skeleton and further reduce RGO. The clay/graphene hydrogel was washed using deionized water for at least 24 h to remove the residual impurities. The purified clay/graphene hydrogel was frozen in liquid nitrogen for 10 min, followed by freeze drying and thermal treatment (400 °C, 1 h).

GA (with MF as sacrificial skeleton) and clay/RGO aerogel (without MF as sacrificial skeleton) was also prepared by the aforementioned method.

2.3 Fabrication of 3D CGAs

A piece of MF was cut into a 3D shape and 3D CGA was prepared by the aforementioned method (**2.2 Fabrication of CGA**).

As shown in Figure 5e, 3D AGA I (30 mm × 30 mm × 20 mm) was fabricated by using a cubic cup shaped MF.

As shown in Figure 5g, 3D AGA II (30 mm × 30 mm × 55 mm) was fabricated by using a pyramidal MF.

2.4 Fabircation of 3D Assembled CGAs

Several MFs were immersed into the above mixed solution by a squeezing procedure for several times. These clay/RGO/MF composites were then kept close together during the preparation process. Finally, 3D assembled CGA with different shapes can be flexibly fabricated.

As shown in Figure 5h-i, 3D AGA III (30 mm × 30 mm × 75 mm) was fabricated by using a pyramidal MF and a cubic cup shaped MF.

As shown in Figure S27, 3D AGA IV (40 mm × 40 mm × 40 mm) with the expanding cross-section was fabricated.

2.5 Construction of 3D Assembled CGA Equipped with p-MF

As shown in Figure S30, Pristine MF (H: 20 cm) was pressed under 230 ^{o}C for 15min to obtain p-MF (H: 2 cm). A piece of p-MF (45 mm × 45 mm × 18 mm) full of water was placed on upper surface of the 3D AGA IV as salt collection system. The p-MF had a hole with the

same size (25 mm × 35 mm) as the inner diameter of the 3D AGA IV to allow sunlight to pass through.

3. Physical Characterizations and Measurement

3.1 Transmission Electron Microscopy (TEM)

Transmission electron microscopy (TEM) was performed on a JEOL JEM-2010 (**Figure 2d-f, Figure S1,** and **Figure S12**).

3.2 Atomic Force Microscope (AFM)

Atomic force microscopy (AFM) images were recorded under ambient conditions using a Digital Instrument Multimode Nanoscope IIIA operating at a tapping mode. Samples were prepared by spin-coating a dispersion of GO (~ 0.3 mg mL^{-1}) onto a freshly cleaved mica surface, respectively. The average size of the GO sheets is ~ 5 μm and the height difference between the steps is ~ 0.9 nm, respectively (**Figure S2**).

3.3 Scanning Electron Microscopy (SEM)

SEM images were obtained on a field-emission scanning electron microscope (Supra 55, ZEISS, Germany) using an accelerating voltage of 10 kV (**Figure 2a-c**, **Figure S3-S5, S9-S11, S13, S18, S32c-d,** and **S41**). All samples were spray-coated with a thin gold layer in vacuum prior to the SEM observations.

3.4 Measurements of Nitrogen Adsorption

Nitrogen adsorption measurements were performed with a Micromeritics TriStar II 3020 ASAP (Micromeritics, USA) to obtain pore properties such as the BET-specific surface area,

pore size distribution, and total pore volume (**Table S1**). Before measurement, the samples were outgassed under vacuum at 100 °C for 10 h until the pressure less than 0.665 Pa.

3.5 X-Ray Diffraction (XRD)

X-ray diffraction (XRD) patterns were recorded using an X´Pert-ProMPD (Holland) D/max-γA X-ray diffractometer with Cu-Kα radiation (λ=0.154056 nm) with a scanning rate of 5° min^{-1} for 2 θ from 5° to 40° (**Figure 2g-i** and **Figure S14**).

3.6 Fourier Transform Infrared (FTIR) Spectroscopy

Fourier transform infrared spectra (FTIR) were measured in the range of 600 - 4000 cm^{-1} with a NICOLET 6700 produced by Thermal Fisher Scientific Corporation (**Figure 2j-l**).

3.7 Thermal Gravimetric Analysis (TGA)

Thermal gravimetric analysis (TGA) was carried out using a thermogravimetric analyzer (TGA8000, PerkinElmer, USA) from 50 to 700 °C at a heating rate of 10 °C min^{-1} under a N_2 atmosphere (**Figure S15).** Before the measurements, all samples were dried in vacuum at 50 °C for 12 h.

3.8 Compression Testing

The compressive tests were performed with a rheometer (MCR 302, Anton Paar, Austria) using a 50 N load cell in the axial-compression testing mode at a strain rate of 10 mm min^{-1}. The cyclic compressions were conducted in air (**Figure 3a-f, Figure S16-17, Table S2-3, and Movie S1**).

3.9 Water Contact Angle Measurement

The sessile drop method was applied to measure the water contact angle using a contact angle instrument (OCA20, Dataphysics, Germany) (**Figure 4a**).

3.10 Measurement of the Solvent Adsorption Capacity

To measure the adsorption capacity of the samples for various solvents, samples were placed inside the solvents for a period of time (~30 s) and then were taken out for measurements. The weights of samples before and after adsorption were measured for calculating the adsorption capacity (**Figure 4b-c, Figure 4f-h, Figure S20, and Table S4-S5**).

3.11 Optical Measurement

The optical transmittance (T) and reflectance (R) spectra were measured in the range of 500 - 2500 nm with a spectrophotometer (UV3600, Shimadzu, Japan) attached to an integrating sphere (ISR-3100) (**Figure 5a**).

3.12 Thermal Conductivity Measurement

The thermal conductivity measurement was performed on a thermal conductivity tester (HFM436, Netzsch, Germany) (**Figure 5b**).

3.13 Evaluation of Solar-driven Steam Generation in Laboratory

Solar simulator (CEL-S500-T5) with an optical filter for the standard AM 1.5 G spectrum was used to simulate sunlight. The water evaporation rate was evaluated by measuring the weight loss of water with an electrical balance (FA 2004, 0.1 mg in accuracy). We use the top projected plane of sample for the light intensity measurement and the radiation intensity is corrected by an optical power meter. An IR camera (FLIR E5xt) was utilized to measure and record the temperature changes. The mass change was measured by an electrical balance and then communicated to a laptop computer in real-time for the evaluation of the evaporation rate

and solar-thermal conversion effciency. The environmental temperature was ~25 °C and the relative humidity was ~ 40%. Note the evaporation rates of all 3D AGA IV equipped with p-MF were calculated by subtracting the evaporation rate of p-MF under one sun illumination (0.38 kg m^{-2} h^{-1}).

3.14 Measurement of Ion Concentration

The ion concentrations of gathered fresh water were measured by the inductively coupled plasma emission spectrometer (ICPE-9820, Shimadzu, Japan) (**Figure 6i-j and Table S7-8**).

4. Supplementary References

[S1] J. Zhong, J. Meng, X. C. Gui, T. Hu, N. Xie, X. Y. Lu, Z. Y. Yang, N. Koratkar. Nanocarbon aerogel complexes inspired by the leaf structure. *Carbon* **2014**, *77*, 637.

[S2] Q. Y. Peng, Y. B. Li, X. D. He, X. C. Gui, Y. Y. Shang, C. H. Wang, C. Wang, W. Q. Zhao, S. Y. Du, E. Z. Shi, P. X. Li, D. H. Wu, A. Y. Cao, Graphene nanoribbon aerogels unzipped from carbon nanotube sponges. *Adv. Mater.* **2014**, 26, 3241.

[S3] X. Wang, L. L. Lu, Z. L. Yu, X. W. Xu, Y. R. Zheng, S. H. Yu. Scalable template synthesis of resorcinol-formaldehyde/graphene oxide composite aerogels with tunable densities and mechanical properties. *Angew. Chem. Int. Ed.* **2015**, *54*, 2397.

[S4] L. Qiu, Z. Liu, L. Y. Chang, Y. Z. Wu, D. Li. Biomimetic superelastic graphene-based cellular monoliths. *Nat. Commun.* **2012**, *3*, 1241.

[S5] C. W. Li, L. Qiu, B. Q. Zhang, D. Li, C. Y. Liu. Robust vacuume/air dired graphene aerogels and fast recoverable shape-memory hybrid foams. *Adv. Mater.* **2016**, *28*, 1510.

[S6] N. Ni, S. Barg, E. G. Tunon, F. M. Perez, M. Miranda, C. Lu, C. Mattevi, E. Saiz. Understanding mechanical response of elastomeric graphene networks. *Sci. Rep.* **2015**, *5*, 13712.

[S7] S.O. Kucheyev, M. Stadermann, S.J. Shin, J.H. Satcher Jr., S.A. Gammon, S.A. Letts, T. van Buuren, A.V. Hamza, Super-compressibility of ultralow-density nanoporous silica. *Adv. Mater.* **2012**, 24, 776.

[S8] H. Lu, C. W. Li, B. Q. Zhang, X. Qiao, C. Y. Liu, Towards highly compressible graphene aerogels of enhanced mechanical performance with polymer. *RSC Adv.* **2016**, 6, 43007.

[S9] H. X. Li, D. D. Jia, M. C. Ding, L. J. Zhou, K. Wang, J. Q. Liu, C. Y. Liu, C. W. Li, Robust 3D graphene/cellulose nanocrystals hybrid lamella network for stable and highly efficient solar desalination. *Sol. RRL* **2021**, 5, 2100317.

[S10] L. Y. Dou, X. X. Zhang, H. R. Shan, X. T. Cheng, Y. Si, J. Y. Yu, B. Ding, Interweaved cellular structured ceramic nanofibrous aerogels with superior bendability and compressibility. *Adv. Funct. Mater.* **2020**, 30, 2005928.

[S11] Z. Y. Wang, X. Shen, M. A. Garakani, X. Y. Lin, Y. Wu, X. Liu, X. Y. Sun, J.-K. Kim. Graphene aerogel/epoxy composites with exceptional anisotropic structure and properties. *ACS Appl. Mater. Interfaces* **2015**, *7*, 5538.

[S12] H. Hu, Z. B. Zhao, W. B. Wan, Y. Gogotsi, J. S. Qiu. Ultralight and highly compressible graphene aerogels. *Adv. Mater.* **2013**, *25*, 2219.

[S13] H. L. Gao, Y. B. Zhu, L. B. Mao, F. C. Wang, X. S. Luo, Y. Y. Liu, Y. Lu, Z. Pan, J. Ge, W. Shen, Y. R. Zheng, L. Xu, L. J. Wang, W. H. Xu, H. A. Wu, S. H. Yu. Super-elastic and fatigue resistant carbon material with lamellar multi-arch microstructure. *Nat. Commun.* **2016**, *7*, 12920.

[S14] J. Kuang, Z. H. Dai, L. Q. Liu, Z. Yang, M. Jin, Z. Zhang. Synergistic effects from graphene and carbon nanotubes endow ordered hierarchical structure foams with a combination of compressibility, super-elasticity and stability and potential application as pressure sensors. *Nanoscale* **2015**, *7*, 9252.

[S15] K. Pang, X. Song, Z. Xu, X. T. Liu, Y. J. Liu, L. Zhong, Y. X. Peng, J. X. Wang, J. Z. Zhou, F. X. Meng, J. Wang, C. Gao, Hydroplastic foaming of graphene aerogels and artificially intelligent tactile sensors. *Sci. Adv.* **2020**, 6, eabd4045.

[S16] X. F. Zhang, G. H. Yang, L. Zong, M. Jiang, Z. Q. Song, C. Ma, T. P. Zhang, Y. X. Duan, J. M. Zhang, Tough, ultralight, and water-adhesive graphene/natural rubber latex hybrid aerogel with sandwichlike cell wall and biomimetic rose-petal-like surface. *ACS Appl. Mater. Interfaces* **2020**, 12, 1378.

[S17] X. Xu, H. Li, Q. Q. Zhang, H. Hu, Z. B. Zhao, J. H. Li, J. Y. Li, Y. Qiao, Y. Gogotsi. Self-sensing, ultra light, and conductive 3D graphene/iron oxide aerogel elastomer deformable in a magnetic field. *ACS Nano* **2015**, *9*, 3969.

[S18] X. Xu, Q. Q.Zhang, M. Hao, Y. Hu, Z. Y. Lin, L. L. Peng, T. Wang, X. X. Ren, C. Wang, Z. P. Zhao, C. Z. Wan, H. L. Fei, L. Wang, J. Zhu, H. T. Sun, W. L. Chen, T. Du, B. W. Deng, G. J. Cheng, I. Shakir, C. Dames, T. S. Fisher, X. Zhang, H. Li, Y. Huang, X. F. Duan. Double-negative-index ceramic aerogels for thermal superinsulation. *Science* **2019**, 363, 723.

[S19] M. W. Peng, Z. Wen, L. J. Xie, J. Cheng, Z. Jia, D. L. Shi, H. J. Zeng, B. Zhao, Z. Q. Liang, T. Li, L. Jiang, 3D printing of ultralight biomimetic hierarchical graphene materials with exceptional stiffness and resilience. *Adv. Mater.* **2019**, 1902930.

[S20] Q. Q. Zhang, X. Xu, H. Li, G. P. Xiong, H. Hu, T. S. Fisher. Mechanically robust honeycomb graphene aerogel multifunctional polymer composites. *Carbon* **2015**, *93*, 659.

[S21] X. C. Gui, J. Q. Wei, K. L. Wang, A. Y. Cao, H. W. Zhu, Y. Jia, Q. K. Shu, D. H. Wu. Carbon nanotube sponges. *Adv. Mater.* **2010**, *22*, 617-621.

[S22] H. P. Cong, X. C. Ren, P. Wang, S. H. Yu. Macroscopic multifunctional graphene-based hydrogels and aerogels by a metal ion induced self-assembly process. *ACS Nano* **2012**, *6*, 2693.

[S23] H. C. Bi, X. Xie, K. B. Yin, Y. L. Zhou, S. Wan, L. B. He, F. Xu, F. Banhart, L. T. Sun, R. S. Ruoff. Spongy graphene as a highly efficient and recyclable sorbent for oils and organic solvents. *Adv. Funct. Mater.* **2012**, *22*, 4421.

[S24] H. W. Liang, Q. F. Guan, L. F. Chen, Z. Zhu, W. J. Zhang, S. H. Yu. Macroscopic-scale template synthesis of robust carbonaceous nanofiber hydrogels and aerogels and their applications. *Angew. Chem., Int. Ed.* **2012**, *51*, 5101.

[S25] J. Y. Hong, E. H. Sohn, S. Park, H. S. Park. Highly-efficient and recyclable oil absorbing performance of functionalized graphene aerogel. *Chemical Engineering Journal.* **2015**, 269, 229-235.

[S26] Y. Li, H. B. Zhang, L. H. Zhang, B. Shen, W. T. Zhai, Z. Z. Yu, W. G. Zheng. One-pot sintering strategy for efficient fabrication of high-performance and multifunctional graphene foams. *ACS Appl. Mater. Interfaces*, **2017**, *9*, 15, 13323.

[S27] J. H. Li, Jingye Li, H. Meng, S. Y, Xie, B. W. Zhang, L. F. Li, H. J. Ma, J. Y. Zhang, M.Yu. Ultra-light, compressible and fire-resistant graphene aerogel as a highly efficient and recyclable absorbent for organic liquids. *J. Mater. Chem. A*, **2014**, *2*, 2934.

[S28] Z. Wang, D. Wang, Z. C. Qian, J. Guo, H. X. Dong, N. Zhao, J. Xu, Robust superhydrophobic bridged silsesquioxane aerogels with tunable performances and their applications. *ACS Appl. Mater. Interfaces* **2015**, *7*, 2016.

[S29] L. Mu, S. D. Yang, B. Hao, P. C. Ma, Ternary silicone sponge with enhanced mechanical properties for oil-water separation. *Polym. Chem.*, **2015**, *6*, 5869.

[S30] Z. L. Yu, G. C. Li, N. Fechler, N. Yang, Z. Y. Ma, X. Wang, M. Antonietti, S. H. Yu. Polymerization under hypersaline conditions: a robust route to phenolic polymer-derived carbon aerogels. *Angew. Chem., Int. Ed.* **2016**, *55*, 14623.

[S31] Q. Zhu, Y. Chu, Z. K. Wang, N. Chen, L. Lin, F. T. Liu, Q. M. Pan. Robust superhydrophobic polyurethane sponge as a highly reusable oil-absorption material. *J. Mater. Chem. A* **2013**, *1*, 5386.

[S32] H. C. Bi, Z. Y. Yin, X. H. Cao, X. Xie, C. L. Tan, X. Huang, B. Chen, F. T. Chen, Q. L. Yang, X. Y. Bu, X. H. Lu, L. T. Sun, H. Zhang. Carbon fiber aerogel made from raw cotton: a novel, efficient and recyclable sorbent for oils and organic solvents. *Adv. Mater.* **2013**, *25*, 5916.

[S33] J. Y. Zhang, Y. H. Cheng, C. J. Xu, M. Y. Gao, M. F. Zhu, L. Jiang, Hierarchical interface engineering for advanced nanocellulosic hybrid aerogels with high compressibility and multifunctionality. *Adv. Funct. Mater.* **2021**, *31*, 2009349.

[S34] C. W. Li, D. G. Jiang, H. Liang, B. B. Huo, C. Y. Liu, W. R. Yang, J. Q. Liu, Superelastic and arbitrary-shaped graphene aerogels with sacrificial skeleton of melamine foam for varied applications. *Adv. Funct. Mater.* **2018**, *28*, 1704674.

[S35] X. Y. Wang, Z. Liu, X. F. Liu, Y. Su, J. Wang, T. T. Fan, X. Ning, S. Ramakrishn, Y. Z. Long, Ultralight and multifunctional PVDF/SiO_2@GO nanofibrous aerogel for efficient harsh environmental oil-water separation and crude oil absorption. *Carbon* **2022**, *193*, 77.

[S36] C. X. Li, J. Yang, P. Pachfule, S. Li, M. Y. Ye, J. Schmidt, A. Thomas, Ultralight covalent organic framework/graphene aerogels with hierarchical porosity. *Nat. Commun.* **2020**, *11*, 4712.

[S37] Y. Shi, C. L. Zhang, R. Y. Li, S. F. Zhuo, Y. Jin, L. Shi, S. Hong, J. Chang, C. Ong, P. Wang, Solar evaporator with controlled salt precipitation for zero liquid discharge desalination. *Environ. Sci. Technol.* **2018**, *52*, 11822.

[S38] X. Su, D. Z. Hao, M. Y. Sun, T. S. Wei, D. K. Xu, X. C. Ai, X. L. Guo, T. Zhao, L. Jiang, Nature sunflower stalk pith with zwitterionic hydrogel coating for highly efficient and sustainable solar evaporation. *Adv. Funct. Mater.* **2021**, 2108135.

[S39] Y. C. Wang, X. Y. Sun, S. Y. Tao, Rational 3D coiled morphology for efficient solar-driven desalination. *Environ. Sci. Technol.* **2020**, *54*, 16240.

[S40] L. Zhu,L. Sun, H. Zhang, H. Aslan, Y. Sun, Y. D. Huang, F. Rosei, M. Yu, A solution to break the salt barrier for high-rate sustainable solar desalination. *Energy Environ. Sci.*, **2021**, *14*, 2451.

[S41] Y. D. Kuang, C. J. Chen, S. M. He, E. M. Hitz, Y. L. Wang, W. T. Gan, R. Y. Mi, L. B. Hu, A high-performance self-regenerating solar evaporator for continuous water desalination. *Adv. Mater.* **2019**, *31*, 1900498.

[S42] S. M. He, C. J. Chen, Y. D. Kuang, R. Y. Mi, Y. Liu, Y. Pei, W. Q. Kong, W. T. Gan, H. Xie, E. Hitz, C. Jia, X. Chen, A. Gong, J. M. Liao, J. Li, Z. J. Ren, B. Yang, S. Das, L. B. Hu, Nature-inspired salt resistant bimodal porous solar evaporator for efficient and stable water desalination. *Energy Environ. Sci.* **2019**, *12*, 1558.

[S43] H. Z. Yao, P. P. Zhang, C. Yang, Q. H. Liao, X. Z. Hao, Y. X. Huang, M. Zhang, X. B. Wang, T. Y. Lin, H. H. Cheng, J. Y. Yuan, L. T. Qu, Janus-interface engineering boosting solar steam towards high-efficiency water collection. *Energy Environ. Sci.* **2021**,*14*, 5330.

[S44] J. X. Xiao, Y. Guo, W. Q. Luo, D. Wang, S. K. Zhong, Y. Yue, C. N. Han, R. X. Lv, J. B. Feng, J. Q. Wang, W. Huang, X. L. Tian, W. Xiao, Y. J. Shen, A scalable, cost-effective and

salt-rejecting MoS_2/SA@melamine foam for continuous solar steam generation. *Nano Energy* **2021**, *87*, 106213.

[S45] J. X. Chen, J. L. Yin, B. Li, Z. Y. Ye, D. L. Liu, D. Ding, F. Qian, N. V. Myung, Q. Zhang, Y. D. Yin, Janus evaporators with self-recovering hydrophobicity for salt-rejecting interfacial solar desalination. *ACS Nano* **2020**, *14*, 17419.

[S46] Y. Xia, Q. F. Hou, H. S. Jubaer, Y. Li, Y. Kang, S. Yuan, H. Y. Liu, M. W. Woo, L. Zhang, L. Gao, H. T. Wang, X. W. Zhang, Spatially isolating salt crystallisation from water evaporation for continuous solar steam generation and salt harvesting. *Energy Environ. Sci.* **2019**, *12*, 1840.

[S47] Y. Shao, J. B. Tang, N. B. Li, T. Y. Sun, L. P. Yang, D. Chen, H. Zhi, D. J. Wang, H. Liu, G. B. Xue, Designing a bioinspired synthetic tree by unidirectional freezing for simultaneous solar steam generation and salt collection. *EcoMat.* **2020**, *2*, e12018.

[S48] C. W. Li, D. G. Jiang, B. B. Huo, M. C. Ding, C. C. Huang, D. D. Jia, H. X. Li, C. Y. Liu, J. Q. Liu, Scalable and robust bilayer polymer foams for highly efficient and stable solar desalination. *Nano Energy* **2019**, *60*, 841.

[S49] N. N. Cao, S. T. Lu, R. Yao, C. X. Liu, Q. Y. Xiong, W. Qin, X. H. Wu, A self-regenerating air-laid paper wrapped ASA 3D cone-shaped Janus evaporator for efficient and stable solar desalination. *Chemical Engineering Journal* **2020**, *397*, 125522.

[S50] N. Xu, J. L. Li, Y. Wang, C. Fang, X. Q. Li, Y. X. Wang, L. Zhou, B. Zhu, Z. Wu, S. N. Zhu, J. Zhu, A water lily-inspired hierarchical design for stable and efficient solar evaporation of high-salinity brine. *Sci. Adv.* **2019**, *5*, eaaw7013.

[S51] W. Zhang, X. Chen, G. Zhang, J. F. Li, Q. H. Ji, C. Z. Hu, Z. J. Ren, H. J. Liu, J. H. Qu, A salt-rejecting anisotropic structure for efficient solar desalination via heat-mass flux decoupling. *J. Mater. Chem. A* **2020**, *8*, 12089.

[S52] C. B. Wang, J. L. Wang, Z. T. Li, K. Y. Xu, T. Lei, W. K. Wang, Superhydrophilic porous carbon foam as a selfdesalting monolithic solar steam generation device with high energy efficiency. *J. Mater. Chem. A* **2020**, *8*, 9528.

[S53] W. C. Xu, X. Z. Hu, S. D. Zhuang, Y. X. Wang, X. Q. Li, L. Zhou, S. N. Zhu, J. Zhu, Flexible and salt resistant janus absorbers by electrospinning for stable and efficient solar desalination. *Adv. Energy Mater.* **2018**, *8*, 1702884.

[S54] W. Wang, Y. Wang, J. Zheng, X. Yu, W. S. Chen, J. H. Li and Y. N. Liu, A vasculatural hydrogel combined with prussian blue for solardriven vapor generation. *J. Mater. Chem. A* **2022**, *10*, 12608-12615.

[S55] L. Wu, Z. C. Dong, Z. R. Cai, T. Ganapathy, N. X. Fang, C. X. Li, C. L. Yu, Y. Zhang, Y. L. Song, Highly efficient three-dimensional solar evaporator for high salinity desalination by localized crystallization. *Nat. Commun.* **2020**, *11*, 521.

[S56] L. Li, N. He, B. Jiang, K. W. Yu, Q. Zhang, H. T. Zhang, D. W. Tang, Y. C. Song, Highly salt-resistant 3d hydrogel evaporator for continuous solar desalination via localized crystallization. *Adv. Funct. Mater.* **2021**, *31*, 2104380.

[S57] S. Chaule, J. Hwang, S. J. Ha, J. Kang, J. C. Yoon, J. H. Jang, Rational design of a high performance and robust solar evaporator via 3D-printing technology. *Adv. Mater.* **2021**, *33*, 2102649.

[S58] Z. X. Liu, B. H. Wu, B. Zhu, Z. G. Chen, M. F. Zhu, X. G. Liu, Continuously producing watersteam and concentrated brine from seawater by hanging photothermal fabrics under sunlight. *Adv. Funct. Mater.* **2019**, *29*, 1905485.

[S59] Z. X. Wang, X. C. Wu, F. He, S. Q. Peng, Y. X. Li, Confinement capillarity of thin coating for boosting solar-driven water evaporation. *Adv. Funct. Mater.* **2021**, *31*, 2011114.

[S60] X. Wu, Y. D. Wang, P. Wu, J. Y. Zhao, Y. Lu, X. F. Yang, H. L. Xu, Dual-zone photothermal evaporator for antisalt accumulation and highly efficient solar steam generation. *Adv. Funct. Mater.* **2021**, *31*, 2102618.

[S61] Y. Sun, Z. B. Zhao, G. Y. Zhao, L. X. Wang, D. Z. Jia, Y. Z. Yang, X. G. Liu, X. Z. Wang, J. S. Qiu, High performance carbonized corncob-based 3D solar vapor steam generator enhanced by environmental energy. *Carbon* **2021**, *179*, 337.

[S62] S. W. Dong, Y. F. Xu, C. J. Wang, C. H. Liu, J. L. Zhang, Y. S. Di, L. Y. Yu, L. F. Dong, Z. X. Gan, Atmospheric water harvester-assisted solar steam generation for highly efficient collection of distilled water. *J. Mater. Chem. A* **2022**, *10*, 1885.

[S63] C. X. Lei, W. X. Guan, Y. H. Guo, W. Shi, Y. Y. Wang, K. P. Johnston, G. H. Yu, Polyzwitterionic Hydrogels for Highly Efficient High Salinity Solar Desalination. *Angew. Chem. Int. Edit.* **2022**, *61*, e202208487.

[S64] X. J. Mu, J. H. Zhou, P. F. Wang, H. Chen, T. T. Yang, S. S. Chen, L. Miao, T. Mori, *Energy Environ Sci.* **2022**, *15*, 3388-3399.
